# Exploring the Potentials of Silver Nanoparticles in Overcoming Cisplatin Resistance in Lung Adenocarcinoma: Insights from Proteomic and Xenograft Mice Studies

**DOI:** 10.1021/acsnano.5c09056

**Published:** 2025-09-26

**Authors:** Tin Yan Wong, Yan Wang, Kenneth Kin Leung Kwan, Yanrong Pan, Alan Ka Lun Lai, Sike Chen, Yao Xiao, Kun Zhou, Long Wu, Sitong Huo, Neng Yan, Henry Lam

**Affiliations:** † Department of Chemical and Biological Engineering, 58207 The Hong Kong University of Science & Technology, Clear Water Bay, Kowloon, Hong Kong 999077, China; ‡ School of Environmental Studies, 12564 China University of Geosciences, Wuhan 430074, China; § Department of Pathology, 71020 The University of Hong Kong, Kowloon, Hong Kong 999077, China; ∥ Department of Ocean Science, The Hong Kong University of Science and Technology, Kowloon, Hong Kong 999077, China; ⊥ Department of Bacteriology, 5228 University of Wisconsin−Madison, Madison, Wisconsin 53706, United States; # St. Jude Children’s Research Hospital, Memphis, Tennessee 38105, United States

**Keywords:** proteomics, silver nanoparticles, cisplatin, resistance, NSCLC

## Abstract

Silver nanoparticles (AgNPs) have shown great potential as therapeutic agents due to their ability to cause apoptotic cell death in cancer cells. However, little knowledge is available regarding the underlying action mechanisms of AgNPs toward multidrug-resistant cancer cells. Herein, we employed quantitative proteomics to investigate the cytotoxic mechanisms of AgNPs and their potential anticancer properties on both cisplatin-sensitive (A549 cells) and -resistant (A549/DDP cells) human lung adenocarcinoma using quantitative proteomics and mice xenograft model approaches. We first performed cytotoxicity tests and found that AgNPs exerted similar cytotoxic effects on A549 and A549/DDP cells. At the proteome level, A549 and A549/DDP cells responded to AgNPs distinctively and similarly by causing cell apoptosis via upregulating RNA metabolism, suppressing the VEGF signaling pathway, repressing p53-mediated pathways, promoting cell cycle arrest, etc. Additionally, we found that AgNPs induced ROS generation and disrupted mitochondrial function and respiration in the A549 and A549/DDP cells. Lastly, animal studies using established mice xenograft models administered with AgNPs showed that AgNPs exhibit similar antitumoral effects on both A549 and A549/DDP-bearing mice. Overall, our investigations showed that AgNPs could effectively induce cell death in lung adenocarcinoma regardless of their sensitivities to cisplatin, suggesting that AgNPs could be potentially used in biomedical aspects as anticancer agents in alleviating the problem of acquired drug resistance in chemotherapy.

## Graphical abstract

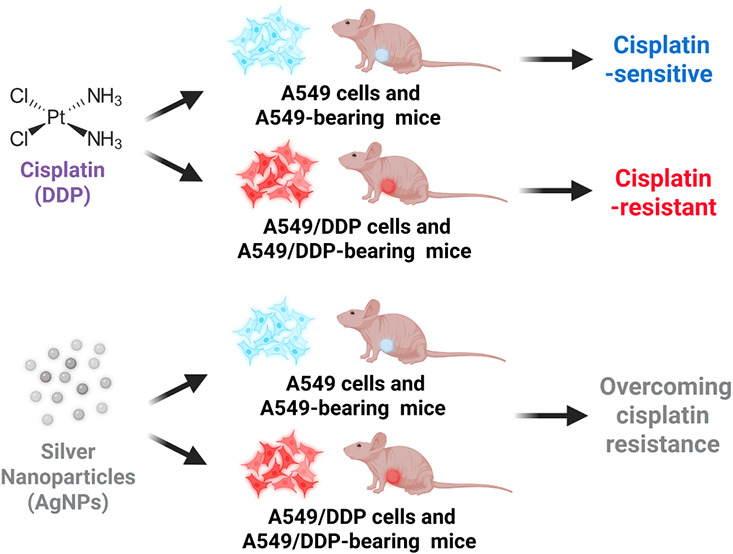

## Introduction

As the leading cause of cancer-related death worldwide, lung cancer remains a significant public health challenge. Lung cancer can be broadly categorized into two main types: small-cell lung carcinoma (SCLC) and non-small-cell lung carcinoma (NSCLC). Of particular concern is NSCLC, which has a high mortality rate and accounts for approximately 85% of all lung cancer cases, which makes it one of the deadliest cancers worldwide.^1^

At present, strategies for treating NSCLC include chemotherapy (commonly employing platinum-based drugs such as cisplatin or carboplatin in combination with etoposide) and radiation therapy.^2^ Among these different treatments, cisplatin (cis-diamminedichloroplatinum­(II), DDP)-based chemotherapy is widely practiced due to its demonstrated high response rates in treating non-small-cell lung carcinoma and great precision in targeting and inhibiting the growth of rapidly dividing cancer cells.^3^ However, it is worth noting that cisplatin can exert severe side effects on humans as it can bind with DNA and subsequently form DNA cross-links, leading to cell cycle arrest and, subsequently, cell apoptosis in a nonspecific way.^4^ More alarmingly, long-term usage of cisplatin can lead to the development of acquired drug resistance in NSCLC, ultimately lowering drug efficacy and further inducing other side effects and toxicity, including nephrotoxicity, neurotoxicity, ototoxicity, and bone marrow suppression.^5,6^ Although reduced drug uptake and increased drug efflux, enhanced DNA repair, and altered cellular targets have been proposed as potential mechanisms for cisplatin resistance in cancer cells, there is still a need for a more comprehensive understanding of the whole picture, as well as better ways on how to bypass drug resistance.

Silver nanoparticles (AgNPs), being one of the most used nanomaterials in biomedical research, have been shown to possess bioactivities such as antibacterial, antifungal, and antiviral activities.^7^ Compared to traditional chemotherapeutic drugs like cisplatin, AgNPs have demonstrated broad-spectrum activity against different cancer cells (including lung cancers) and lower toxicity toward normal cells.^7^ Previous studies have shown the efficacy of AgNPs as anticancer agents in combating multidrug-resistant cancer,^8,9^ as AgNPs have been known to exert excellent antiproliferative activity in different human cell types.^10^ Their cytotoxic mechanisms are thought to be associated with their unique ability to generate reactive oxygen species (ROS), which can cause imbalance of ROS homeostasis and eventually cell death. Besides, AgNPs can also interact with DNA molecules and induce DNA damage in cancer cells, leading to disruptions in DNA replication and transcription processes and ultimately triggering cell cycle arrest and apoptosis. AgNPs can also interfere with various signaling pathways involved in cancer cell survival and proliferation. These properties make AgNPs a suitable candidate as anticancer agents. Despite these findings, limited studies have reported the underlying mechanism of how AgNPs exert cytotoxicity on drug-resistant cancer cells at a systematic level, and this should be thoroughly evaluated.

This study aimed to elucidate the underlying action mechanisms of cisplatin and AgNPs on NSCLC models using quantitative proteomics and mice xenograft models (Scheme 1). Human lung adenocarcinoma A549 cells (cisplatin-sensitive) and human lung adenocarcinoma A549/DDP cells (cisplatin-resistant) were chosen as the *in vitro* research models. We first perform comparative studies to investigate the impact of AgNPs on cisplatin-sensitive and -resistant NSCLC cells, aiming to evaluate the anticancer potential of AgNPs and assess their effectiveness in overcoming cisplatin resistance. Then, we compared the proteome alterations of cisplatin-sensitive and cisplatin-resistant human lung adenocarcinoma cells. We pinpointed the differentially expressed proteins induced by AgNPs and cisplatin, followed by bioinformatic analysis. Besides, chemical and biological assays, including cell viability tests, mitotoxicity tests, and intracellular ROS measurements, were also conducted to supplement and support our proteomics results. Furthermore, we conducted *in vivo* experiments using established A549 and A549/DDP-bearing mice to examine the anticancer efficacy and safety of AgNPs in animals. To the best of our knowledge, this is the first systematic, mechanistic, and comparative study on the cytotoxicity and anticancer potentials of AgNPs on cisplatin-sensitive and -resistant lung adenocarcinoma by means of proteomic and xenograft studies. Our results shed light on the underlying action mechanisms of how AgNPs overcome cisplatin resistance in lung adenocarcinoma, providing new opportunities for AgNPs as potential anticancer agents in clinical and biomedical applications.

**Scheme 1:**
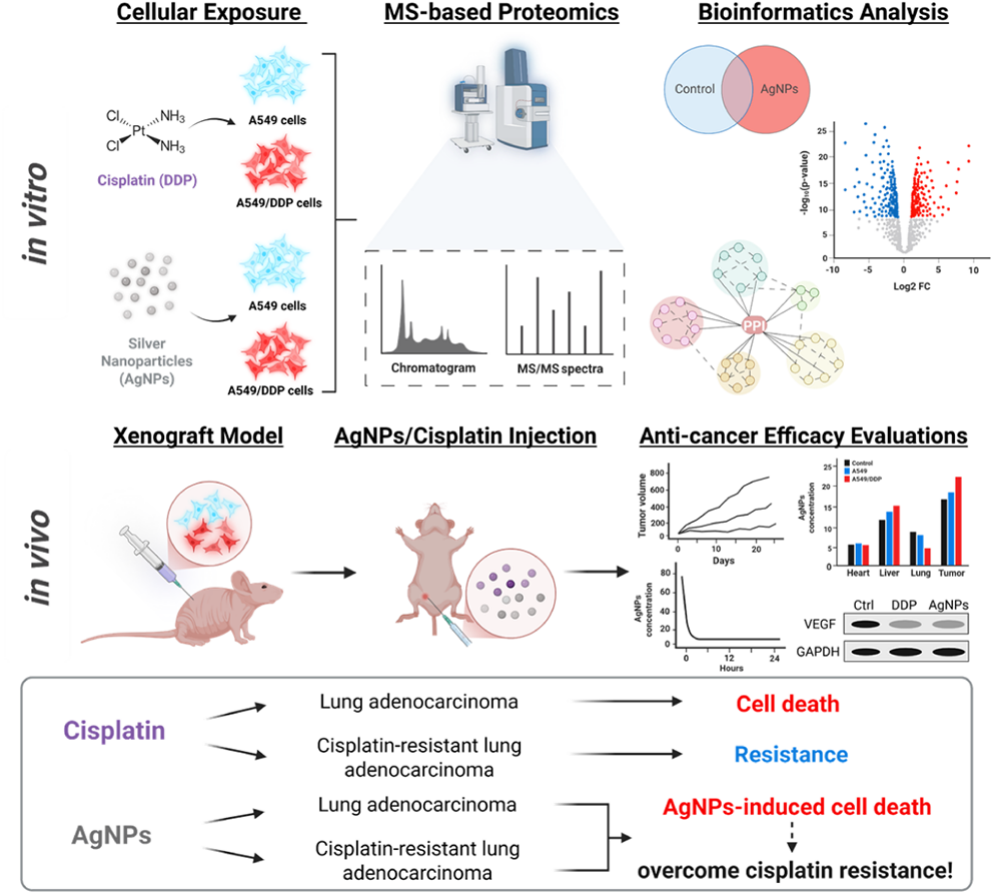
Schematic Illustration of the Experimental Design for the Studies of Anticancer Efficacy by AgNPs in Lung Adenocarcinoma, Both *In Vitro* and *In Vivo*

## Results and Discussion

### Characterization of the Synthesized AgNPs

After synthesizing and purifying AgNPs, their physiochemical properties, including particle size (primary particle diameter) and morphology, were characterized by DLS and transmission electron microscopy (TEM). As shown in Figure 1a, synthetic AgNPs were evenly dispersed, and their shapes were spherical. Figure 1b shows that the average primary particle diameter of AgNPs was 23.45 nm, with zeta potentials lying on −20.33 mV. Moreover, to characterize the surface functional groups of our synthesized AgNPs, FTIR was applied, and the obtained results (Figure 1c) showed the presence of citrate, suggesting that the citrate group was successfully coated on AgNPs with reference to Ramalingam et al.^11^ FTIR results (Figure 1d) showed that the main peak of AgNPs was at 400 nm.

**Figure 1:**
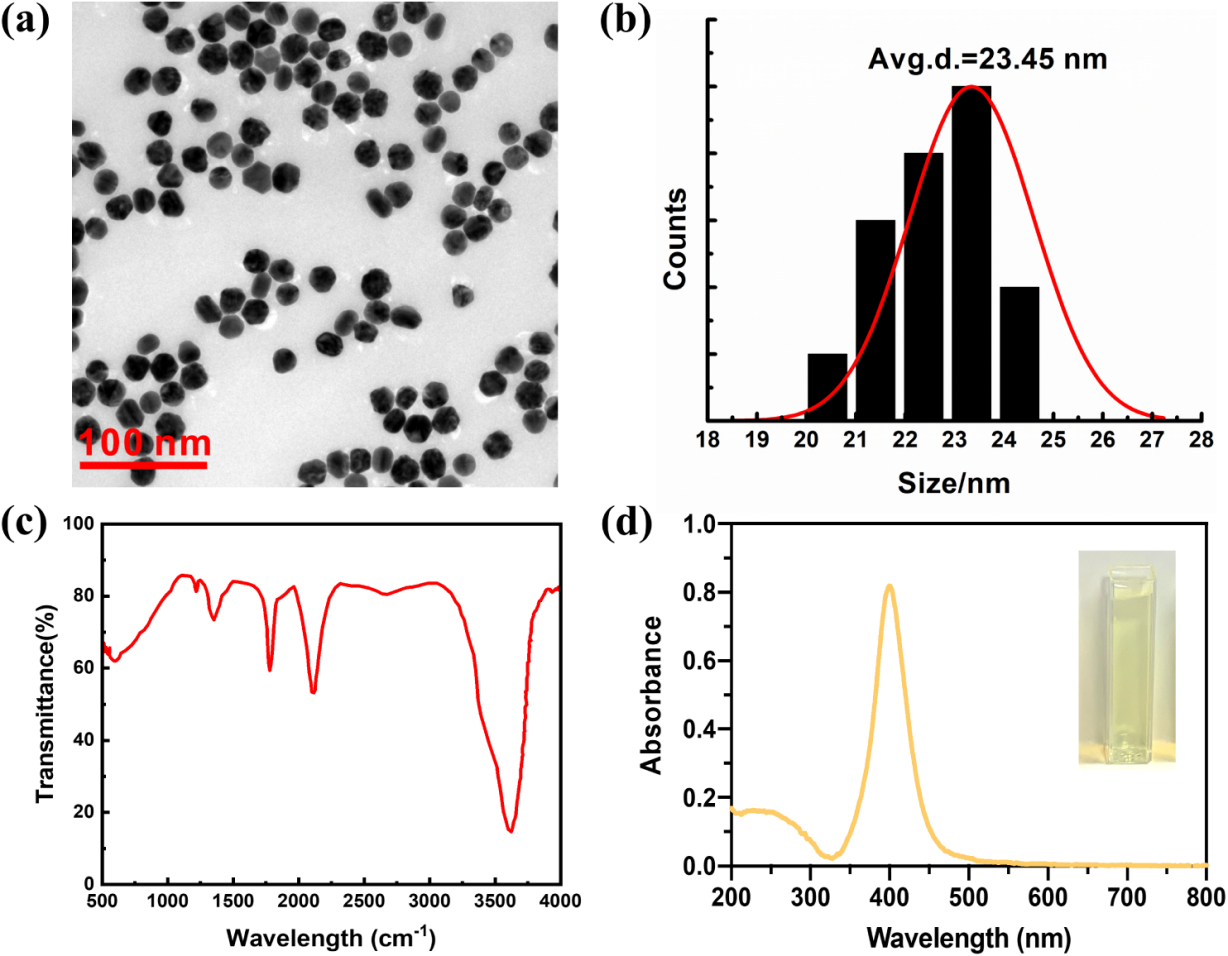
Physiochemical properties of AgNPs. (a) Transmission electron microscopy (TEM) images, (b) particle size measured by dynamic light scattering (DLS), (c) IR spectra, and (d) UV absorption.

The dissolution kinetics of AgNPs in cell culture medium were first examined (as shown in Figure 1). With increasing exposure time, the concentration of AgNPs progressively increased. At dissolution equilibrium, AgNPs released approximately 55.49 μg L^–1^ Ag^+^, which was significantly lower than the Ag^+^ concentration observed in deionized water (208.30 μg L^–1^). Furthermore, AgNPs exhibited faster aggregation kinetics in deionized water compared to those in RPMI 1640 medium (Figure 2). Upon reaching aggregation equilibrium, the particle size of AgNPs in deionized water increased to ∼401.09 nm, whereas those in cell culture medium remained at ∼155.49 nm. This suggests that AgNPs maintain greater stability in cell culture medium under these conditions.

**Figure 2:**
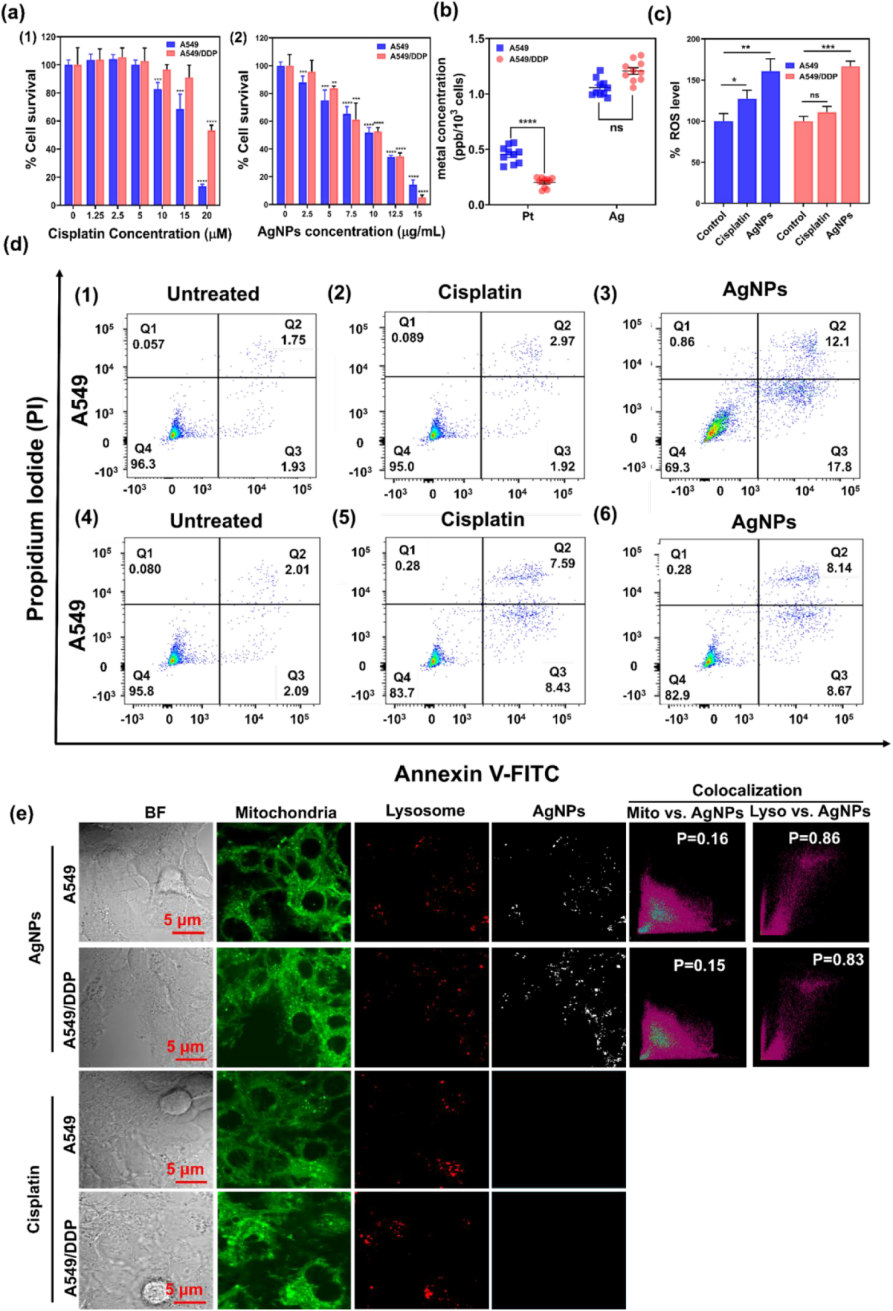
Cellular effects of human lung adenocarcinoma cells on AgNPs and cisplatin. (a) Cytotoxicity of A549 and A549/DDP cells exposed to (1) 10 μM cisplatin and (2) 2.5 μg/mL AgNPs for 24 h and measured by MTT assay. Data are the mean ± SEM of *n* = 5. *p < 0.05, **p < 0.01, ***p < 0.005, ****p < 0.001 (in comparison to respective untreated control by *t*-test). (b) Intracellular concentrations of silver (Ag) and platinum (Pt) in A549 and A549/DDP cells exposed to 10 μM cisplatin and 2.5 μg/mL AgNPs for 24 h, respectively. Results are expressed in ppb/10^3^ cells. Mean ± SEM of *n* = 10. *p < 0.05, **p < 0.01, ***p < 0.005 (in comparison to respective untreated control by *t*-test). (c) Intracellular ROS measurements of A549 and A549/DDP cells treated with cisplatin or AgNPs. (d) Live/dead cell viability of human lung adenocarcinoma cells exposed to cisplatin and AgNPs. A549 cells exposed to (1) nothing, (2) 10 μM cisplatin, (3) 2.5 μg/mL AgNPs, and A549/DDP cells exposed to (4) nothing, (5) 10 μM cisplatin, and (6) 2.5 μg/mL AgNPs for 24 h. (e) Confocal microscopy images illustrating the intracellular distribution of AgNPs and their colocalization with mitochondria (Mito) and lysosomes (Lyso).

Additionally, we investigated the bioaccumulation of Ag in A549 and A549/DDP cells following 24-h exposure to AgNPs or Ag^+^. After 24 h of AgNPs exposure, the intracellular Ag content was measured as 1.10 ± 0.11 ppb Ag per 10^3^ cells in A549 cells and 1.20 ± 0.08 ppb Ag per 10^3^ cells in A549/DDP cells (Figure S3). In contrast, exposure to Ag^+^ resulted in significantly lower bioaccumulation: 0.16 ± 0.03 ppb Ag per 10^3^ cells in A549 cells and 0.17 ± 0.03 ppb Ag per 10^3^ cells in A549/DDP cells. These results indicate that AgNPs are internalized by cells far more effectively than free Ag^+^ under identical exposure conditions, which can be attributed to their distinct internalization pathways (with AgNPs via endocytosis, while Ag^+^ via transporters).^12^

### Sensitivity of A549/DDP Cells and A549 Cells to AgNPs and Cisplatin

In this study, our initial focus was on examining the cytotoxic effects of AgNPs and cisplatin on human adenocarcinoma cells for 24 h. The aim was to determine the optimal concentrations of these substances for subsequent proteomics studies. To ensure accurate results, we used FBS-free RPMI 1640 medium to prepare various concentrations of AgNPs or cisplatin for cellular exposure. This approach avoided any potential interactions between AgNPs/cisplatin and the proteins present in the FBS medium. Figure 2a shows that cisplatin exhibited minimal cytotoxicity in both adenocarcinoma cell lines when its concentration was below 10 μM. However, significant cytotoxic effects were observed in A549 cells at concentrations higher than 10 μM of cisplatin. In the case of A549/DDP, cytotoxic effects were only significant at cisplatin concentrations exceeding 20 μM. These findings indicate that A549 cells are more sensitive to cisplatin than A549/DDP cells. Interestingly, AgNPs demonstrated similar cytotoxicity in both A549/DDP and A549 cells. Concentrations of AgNPs below 2.5 μg/mL had negligible effects on the viability of both lung adenocarcinoma cell lines. However, a dose-dependent decrease in cell viability was observed for AgNPs. Based on this data, we selected 10 μM cisplatin and 2.5 μg/mL AgNPs as sublethal concentrations for cellular exposure in subsequent experiments involving both A549/DDP and A549 cells. These concentrations ensured that cell survival rates remained around or above 80% for both cell lines. Using these concentrations, we could observe the regulated cellular responses to AgNPs exposure while minimizing the presence of dead cells in the proteomics samples. Consistent with the live/dead cell viability test as shown in Figure 2d, which suggested that AgNPs could induce similar population numbers of apoptotic cell populations in A549 and A549/DDP cells, while cisplatin induced apoptosis in A549/DDP to a lesser extent compared to A549/DDP.

It is also interesting to know the internalization pathways of AgNPs, as internalization is one of the important factors governing the sensitivities of A549 and A549/DDP to AgNPs. Therefore, AgNPs have been further explored by exposing the cells to different endocytosis inhibitors.^13^ Monodansylcadaverine (MDC), filipin complex (FC), 5-(N-ethyl-*N*-isopropyl)-amiloride (EIPA), and sodium azide (NaN_3_) were used to block the clathrin- and caveolin-dependent endocytosis, macropinocytosis, and energy-dependent endocytosis, respectively. MDC, FC, and NaN_3_ were used to block the clathrin- and caveolin-dependent endocytosis and energy-dependent endocytosis, respectively. For the internalized AgNPs, clathrin-dependent endocytosis was the main endocytosis pathway since MDC treatment exerted the highest inhibition efficiency (more than 75%, Figure S4). Therefore, the AgNPs were more likely internalized through the clathrin-dependent endocytosis, which was similar to the previous studies.^14^ It is worth mentioning that, unlike cisplatin, which only enters the cells via membrane transporters, AgNPs could enter the cells via both endocytosis and membrane transporters, which makes AgNPs have a lower tendency to induce multidrug resistance compared to traditional chemotherapeutic drugs.^15^

It should be noted that whether cancer cells will also develop resistance to nanomedicine drugs, as they do with other chemotherapies, remains an open question. Thanks to the versatile and unique physicochemical properties of nanomaterials,^16^ some studies have shown that nanomedicine drugs have the potential to combat drug resistance in various types of cancers^17^ by improving pharmacokinetics, precisely targeting tumor cells, reducing side effects, and even reversing drug resistance.^18^ AgNPs, among the most commonly used nanomaterials, exhibit promising antibacterial, antiviral, and anticancer activities through their unique modes of action,^19^ making them an excellent candidate for investigating strategies to combat multidrug-resistant cancers.

### Intracellular Uptake and Biodistribution of AgNPs and Cisplatin

We measured the intracellular metal concentrations by ICP-MS after the cells were exposed to AgNPs or cisplatin for 4 h. From the results, Ag levels in both lung adenocarcinoma cells after exposure to AgNPs were similar, at around 1.1 μg/L/10^3^ cells/mL and 1.2 μg/L/10^3^ cells/mL in A549/DDP and A549 cells, respectively. In contrast, there are significant differences in Pt levels between A549/DDP and A549 cells after exposure to cisplatin. For A549 cells, the Pt concentration is around 0.5 μg/L/10^3^ cells/mL, about five times higher than that in A549/DDP (0.1 μg/L/10^3^ cells/mL). This suggests that the cisplatin resistance of A549/DDP can be at least partially traced to decreased influx or increased drug efflux, consistent with previous studies.^20,21^ The ICP-MS results are shown in Figure 2b.

Furthermore, we investigated the biodistribution of AgNPs in human lung adenocarcinoma cells. Figure 2e shows that AgNPs were internalized and accumulated in the lysosomes of both A549 and A549/DDP cells in a similar way. On the other hand, for the biodistribution of cisplatin, previous studies have shown that cisplatin is sequestered in lysosomes specifically,^22,23^ and lysosomal exocytosis plays a crucial role in cisplatin resistance.^24^

### Intracellular ROS Levels upon Cisplatin and AgNPs Treatment

As disruption to ROS homeostasis is known to be a cytotoxicity mechanism for both cisplatin and AgNPs, we measured the ROS generation in both cell types upon cisplatin and AgNPs treatment in comparison to their corresponding untreated cells using CM-H_2_DCF-DA as an ROS indicator. As shown in Figure 2c, AgNPs could significantly induce ROS generation by around 60% in both A549 and A549/DDP cells exposed to AgNPs, compared to untreated cells. For cisplatin-treated A549 cells, around 25% higher ROS levels were observed than in untreated cells, but there was no significant change in ROS levels in cisplatin-treated A549/DDP cells. From our proteomics data, we also found that cellular response to stress could be triggered in both A549 and A549/DDP cells in response to AgNPs, implying that AgNPs could induce higher stress levels and thus higher ROS levels in lung cancer cells (Figure 4c2,c4).

### Mitotoxic Effects of Cisplatin and AgNPs on Human Lung Adenocarcinoma Cells

The mitochondria have been identified as one of the major target organelles for AgNP-induced toxicity, and previous studies have shown that mitochondrial reactive oxygen species (mtROS) can be produced upon AgNP exposure, causing mitochondrial dysfunction.^25^

We monitored the mitochondrial morphology and function to further investigate the biological responses of A549 and A549/DDP cells upon exposure to cisplatin and AgNPs. First, we compared the mitochondrial network morphologies of two types of cells. A distinct change in the mitochondrial network morphology was observed when A549 cells were exposed to cisplatin and AgNPs. Unlike the tubular mitochondrial network in untreated A549 cells, the mitochondria in cells exposed to cisplatin and AgNPs appeared fragmented (Figure 3a). Similarly, exposure to AgNPs also induced a fragmented mitochondrial network morphology in A549/DDP cells. In contrast, the mitochondrial network in A549/DDP cells remained tubular even after exposure to cisplatin, resembling the morphology observed in untreated cells (Figure 3e).

**Figure 3:**
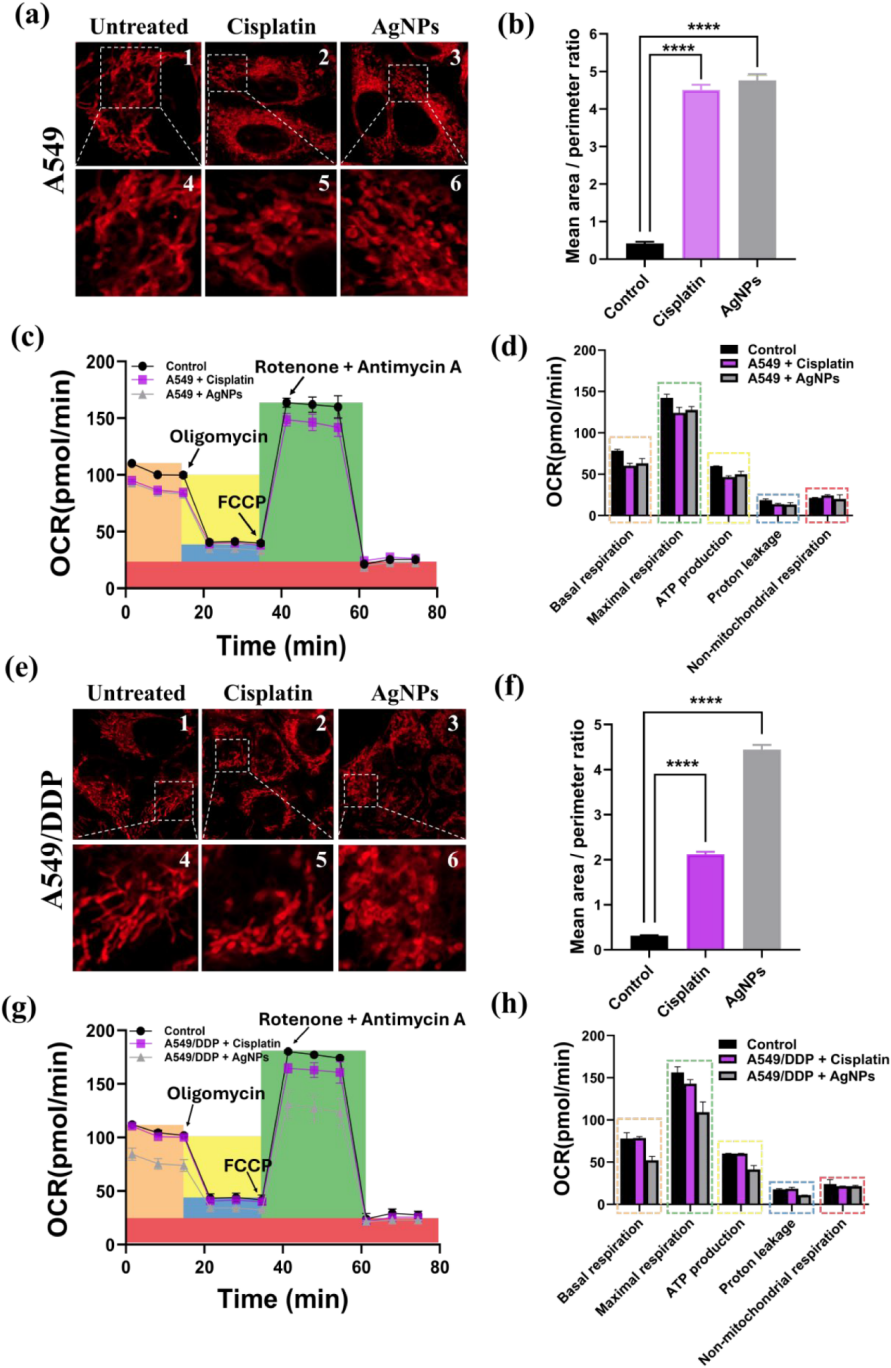
Mitotoxic effects of cisplatin and AgNPs on human lung adenocarcinoma cells. (a,e) Confocal images of mitochondria in (a) A549 cells and (e) A549/DDP cells exposed to (1) nothing, (2) 10 μM cisplatin, and (3) 2.5 μg/mL AgNPs, and their respective enlarged views of the white dashed region as shown in (4), (5), and (6). (b,f) Mitochondrial interconnectivity was calculated using the average area/perimeter. Data are the Mean ± SEM of *n* = 20. *p < 0.05, **p < 0.01, ***p < 0.005, ****p < 0.001 (in comparison to control groups by *t*-test). The scale bar is 5 μm. (c,g) Mitochondrial respiration of lung adenocarcinoma cells exposed to 2.5 μg/mL AgNPs or 10 μM cisplatin compared to respective control groups, indicated by oxygen consumption rate (OCR), was assessed by the Seahorse Mito Stress assay. OCR of (c) A549 cells and (g) A549/DDP cells under different treatments and their respective detailed OCR (d,h) as plotted in bar charts.

Moreover, we also measured the interconnectivity of mitochondria, as shown in Figure 3b,f. We observed that AgNP exposure led to low interconnectivity in both A549 and A549/DDP cells, while cisplatin exposure reduced the interconnectivity in A549/DDP cells but not in A549 cells.

Second, we employed the Seahorse XFp analyzer to comprehensively assess the mitochondrial function of both A549 and A549/DDP cells, with and without cisplatin and AgNPs exposure. The Seahorse XFp analyzer measures parameters such as basal respiration, maximal respiration, ATP production, proton leak, and nonmitochondrial respiration. Figure 3c,g shows the oxygen consumption rate traced from extracellular flux analysis after the A549 and A549/DDP cells were exposed to cisplatin and AgNPs, respectively. For the A549 cells, both cisplatin and AgNPs treatments decreased the basal respiration, maximal respiration, ATP production, proton leak, and nonmitochondrial respiration of A549 cells compared to that of untreated cells (Figure 3d). Similarly, AgNPs exposure also decreased basal respiration (52.3 ± 4.6 pmol/min/10^6^ cells), maximal respiration (109.2 ± 12.2 pmol/min/10^6^ cells), ATP production (41.4 ± 4.6 pmol/min/10^6^ cells), proton leak (11.0 ± 40.1 pmol/min/10^6^ cells), and nonmitochondrial respiration (21.6 ± 0.9 pmol/min/10^6^ cells) of A549/DDP cells. However, cisplatin appeared to exert limited effects on the mitochondrial activities in A549/DDP cells, showing no significant difference in any measured parameters (Figure 3h). These findings suggest that AgNPs are mitotoxic for both cell types, while the effects of cisplatin are more specific to A549 cells and less pronounced in A549/DDP cells.

From observations in mitochondrial morphology and function, we conclude that cisplatin induces less ROS production and fewer morphological changes in mitochondria in A549/DDP cells in comparison to those in A549 cells, which may explain why cisplatin has limited cytotoxicity in A549/DDP. On the contrary, AgNPs have similar and significant effects on ROS production and mitochondrial morphology and function in both types of cells. These results are in good agreement with the proteomics data, which are shown in the PPI interaction (Figure 4c2,c4), that AgNPs could trigger an oxidative stress response. Apart from the protein–protein interaction analysis, we also observed from Gene Ontology (GO) analysis that mitochondria are the third most enriched organelle (besides cytoplasm and nucleus), as in Figure S6b,d.

**Figure 4:**
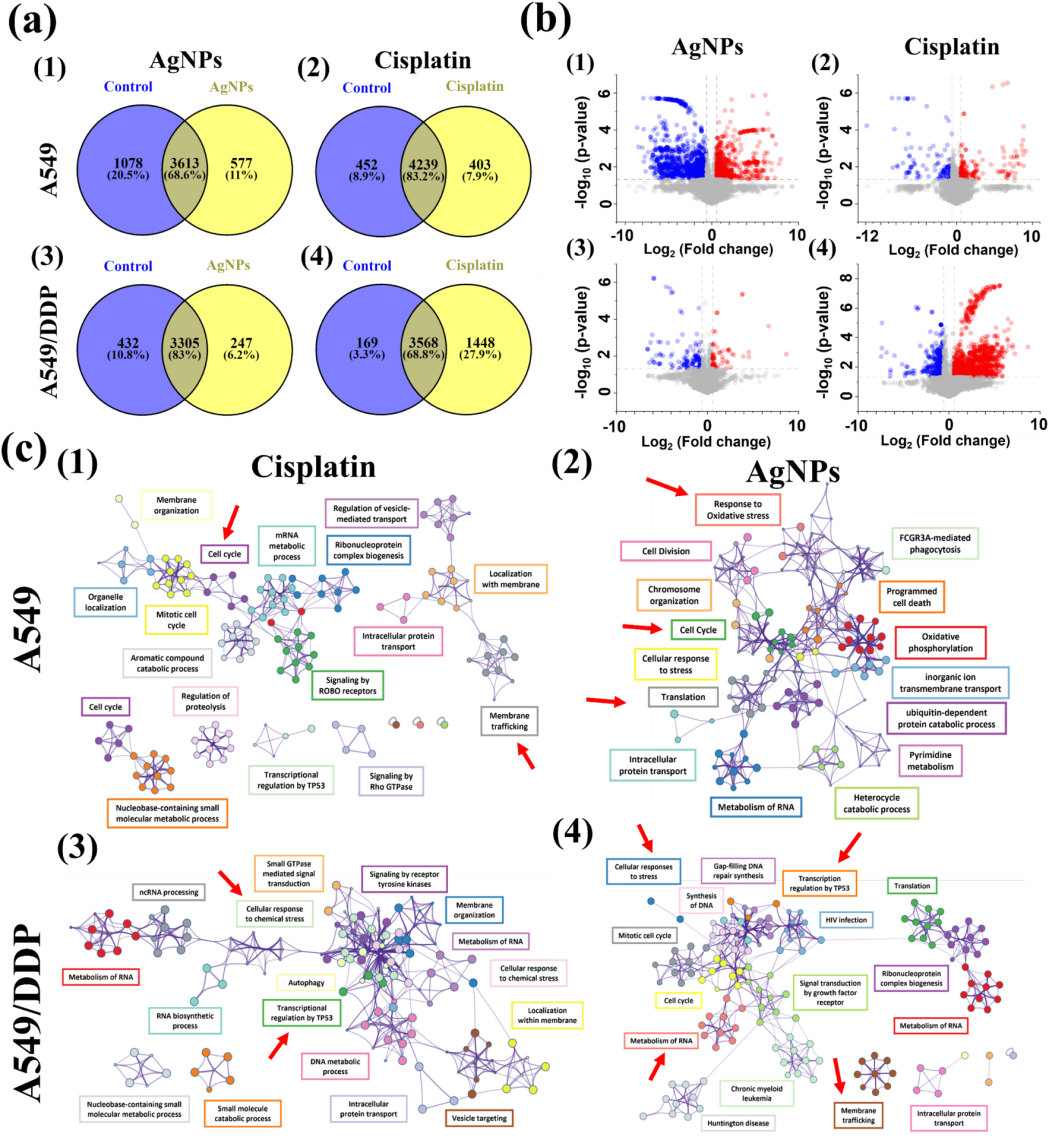
Proteomics analysis of human lung adenocarcinoma cells upon cisplatin and AgNPs treatments. (a) Venn diagrams for the proteome comparison of (1) 2.5 μg/mL AgNPs-treated A549 cells, (2) 10 μM cisplatin-treated A549 cells, (3) 2.5 μg/mL AgNPs-treated A549/DDP cells, and (4) 10 μM cisplatin-treated A549/DDP cells compared to their corresponding untreated cells after 24 h. (b) Volcano plots of differentially expressed proteins in (1) 2.5 μg/mL AgNPs-treated A549 cells compared to the untreated A549 cells, (2) 10 μM cisplatin-treated A549 cells compared to the untreated A549 cells, (3) 2.5 μg/mL AgNPs-treated A549/DDP cells compared to the untreated A549/DDP cells, and (4) 10 μM cisplatin-treated A549/DDP cells compared to the untreated A549/DDP cells, respectively. Differentially expressed proteins are defined as significant if fold change >1.5 or < −1.5 and *p* < 0.05, corresponding to the regions marked with gray dashed lines. Red dots represent upregulated proteins, blue dots represent downregulated proteins, and gray dots represent proteins identified with no significant changes. (c) Protein–protein interaction networks of the differentially expressed proteins (DEPs) of (1) 10 μM cisplatin- and (2) AgNPs-treated A549 cells compared to its untreated A549 cells, and (3) 10 μM cisplatin- and (4) AgNPs-treated A549/DDP compared to its untreated cells were analyzed with Metascape and visualized with Cytoscape (v3.1.2) with a “force-directed” layout. Enrichment *p*-values were generated by Metascape by using cumulative hypergeometric distributions.

### Proteome Profiling of A549/DDP and A549 Cells

To investigate the cellular responses triggered by cisplatin and AgNPs in human lung adenocarcinoma cells, both cell lines were treated with either 10 μM cisplatin or 2.5 μg/mL AgNPs for 24 h, considering that the cell viabilities under these concentrations were all above 80%. MS-based label-free quantitative (LFQ) proteomic analysis was employed to identify the differentially expressed proteins (DEPs) upon treatments compared to the untreated controls.

For A549/DDP cells, 3552, 5016, and 3737 proteins were identified in AgNPs-treated, cisplatin-treated, and untreated control groups, respectively. For A549 cells, 4190, 4642, and 4691 proteins were identified in AgNPs-treated, cisplatin-treated, and untreated control groups, respectively. Good data quality was shown by the good reproducibility of the quantitative measure NSAF with Pearson’s correlation between 0.981 and 0.891 within biological replicates (Figure S5).

To identify the differentially expressed proteins (DEPs), volcano plots were plotted with the criterion of fold changes (>1.5 or <−1.5) against *p*-values (<0.05). In A549/DDP cells, 163 proteins were significantly altered, with 50 being upregulated and 113 being downregulated in the AgNPs-treated group, whereas 1195 proteins were significantly altered, with 895 being upregulated and 300 being downregulated in the cisplatin-treated group. In A549 cells, 1266 proteins were significantly altered, with 482 being upregulated and 784 being downregulated in the AgNPs-treated group, whereas 236 proteins were significantly altered, with 108 being upregulated and 128 being downregulated in the cisplatin-treated group (Figure 4a).

To further highlight the processes involved, we employed gene ontology (GO) and pathway enrichment analysis on the DEPs of human lung adenocarcinoma upon AgNPs treatment using DAVID and Metascape, respectively. Figure S6a–d showed the affected cellular processes upon cisplatin treatment in A549 cells by gene ontology (GO) analysis, and Figure S7a–d showed the enriched pathways. The protein–protein interaction networks in all experimental groups are shown in Figure 4c1–c4.

### Altered Pathways in Cisplatin-Sensitive and -Resistant A549 Cells Exposed to Cisplatin

To better understand the proteins involved in the perturbed pathways upon exposure, bioinformatic tools, including KEGG (Kyoto Encyclopedia of Genes and Genomes) and GO (Gene Ontology), were applied. Figure S6a,c shows the GO analysis of the perturbed process of A549 and A549/DDP cells exposed to cisplatin, and Figure S7a,c shows the enriched pathways. Tables S1 and S2 show the altered pathways related to cisplatin-induced cytotoxicity in both A549 and A549/DDP cells.

From our above experimental results, it was found that A549 and A549/DDP have different sensitivities toward cisplatin. Our proteomics results also showed that it responded to cisplatin differently with respect to altered pathways. First, we observed upregulation of the metabolism of RNA-related proteins, including AKT1, NCBP1, POLR2H, and PRKCD in A549 cells and CDC5L, DDX1, ERCC2, and FBL in A549/DDP cells. Yang et al. also found similar results when they attempted to identify the signature of resistance-specific by the mRNA expression profile using microarray.^26^ More importantly, processes involving long noncoding (lnc) RNA synthesis were also observed, with the upregulation of NCBP1, RRP9, PRORP, and NSA2 in A549 cells and DDX1, ERCC2, FBL, and EXOSC9 in A549/DDP cells. The importance of lncRNA in regulating the cell cycle was highlighted.^27^

Cisplatin is expected to trigger p53-related pathways in cells, promoting tumor suppression. TP53 is a key transcription factor regulating cell proliferation. In A549 cells, TP53-associated proteins like AKT1, FANCD2, and MTOR were all downregulated, implying that repressing TP53-related proteins could facilitate cell apoptosis. While for A549/DDP cells, proteins participating in the regulation of TP53, such as CASP6, CHD4, ERCC2, and COX6B1, were upregulated, indicating that TP53 pathways were positively regulated and cell proliferation was promoted. It is worth noting that in cisplatin-resistant cells, TP53 (p53) is usually mutated and loses its function in mediating cell apoptosis, which suggests that the significant upregulation of TP53-related pathways in cisplatin-resistant cells may not have the intended effect.^28^

It is largely known that cisplatin can cross-link with DNA to form DNA adducts, which could potentially cause DNA damage and trigger the DNA repair mechanism.^20^ In A549 cells, it was found that cisplatin exposure significantly altered proteins that participated in DNA damage response, with the downregulation of DNA repair proteins such as DNMT1, POLA1, and PRIM1. Negative regulation of DNA repair could cause DNA damage and later cell apoptosis. However, in A549/DDP cells, DNA repair proteins, including BAK1, CBL, CETN1, and CRIP1, were upregulated. We hypothesized that it is because of the significantly lower intracellular cisplatin level in A549/DDP cells compared to A549 cells, and thus, the DNA repair mechanism could be promoted only to a lesser extent. Without promoting the DNA repair response, A549 cells are expected to be more susceptible to cell apoptosis.

In addition, cisplatin is known to affect the cell cycle of A549 cells by arresting the G2/M phase, repressing the synthesis of DNA, and subsequently causing cell apoptosis.^21^ In A549 cells, proteins involved in the cell cycle after cisplatin exposure, such as CDK6, RAD21, KIF23, and SUN2, were downregulated, indicating that cisplatin could effectively induce cell cycle arrest. However, in A549/DDP cells, DNA synthesis is promoted as proteins such as CDC27, CDK2, CDK6, and CDKN1B were upregulated, suggesting that the lack of ability for cell cycle arrest in cisplatin-resistant A549 cells is probably due to the promotion of DNA synthesis. Several studies have also mentioned that the abrogation of cell cycle arrest is the main reason for cisplatin resistance.^29–31^

Interestingly, for A549/DDP cells exposed to cisplatin, several proteins associated with membrane trafficking, such as AP2A2, CAPZB, and CUX1, have been downregulated. This result suggested that negative regulation of these membrane trafficking proteins could help reduce the influx of cisplatin and thus alleviate the cytotoxicity of cisplatin to A549/DDP cells. On the other hand, membrane trafficking proteins such as PPP1R10, BAG3, and TMED3 were upregulated in A549 cells exposed to cisplatin, suggesting that positive regulations of membrane trafficking proteins could help with the cisplatin influx, causing cytotoxic effects to A549 cells. Our data is consistent with the notion that A549/DDP cells overcome platinum-based drug resistance by altering membrane protein trafficking.^32,33^

### Altered Pathways in Cisplatin-Sensitive and -Resistant A549 Cells Exposed to AgNPs

The cytotoxicity of AgNPs toward both cisplatin-sensitive and -resistant lung cancer cell lines is at a similar level, as measured by the MTT cell viability test. Interestingly, the proteome changes of both cell lines induced by AgNPs are also very similar. The downregulations of proteins involved in the VEGFA-VEGFR2 signaling pathway were observed in both A549 and A549/DDP cells treated with AgNPs. Among proteins involved in the VEGFA-VEGFR2 signaling pathway, FAS, ARF4, and CFL1 were significantly downregulated by −2.722, −1.763, and −0.668 in AgNP-treated A549 cells treated with AgNPs, while ARF4 and FN1 were downregulated by −0.821 and −1.530 in AgNP-treated A549/DDP cell, respectively. The VEGF pathway is a well-known pathway in regulating angiogenesis and apoptosis. Downregulation of proteins involved in the VEGF signaling pathway could suppress angiogenesis and thus promote cell apoptosis. Antiangiogenic properties of AgNPs have been previously reported.^34^

Perturbation of several pathways that could induce cell apoptosis, including cell cycle, metabolism of RNA, regulation of TP53, and programmed cell death, was also observed in both A549 and A549/DDP cells upon AgNPs treatments. As discussed above, the metabolism of RNA is crucial in biological processes, especially cell apoptosis in this study. Upregulation of proteins engaged in RNA metabolism, including CLNS1A and CSTF1 in A549 cells, and POLR2A, PSMA3, and RPL13 in A549/DDP cells, was observed upon AgNPs treatment. Upregulation of RNA proteins prepares for subsequent pathways, such as cell cycle arrest and TP53 regulation. Furthermore, AgNPs are known to play an important role in inducing apoptosis via cell cycle arrest.^35,36^ Negative regulation of proteins that take part in the cell cycle process has been observed upon AgNPs exposure, including CDK2 and ATRX in A549 cells and KIF2A, CKS1B, MCM3, and MCM7 in A549/DDP cells, respectively. This result suggests that inhibition of protein expression in the cell cycle could induce cell cycle arrest and later cell apoptosis. Besides, we observed that inhibition of TRAF2,^37^ STK26,^38^ CYS,^39^ and CRK^40^ in A549/DDP cells and COX5B,^41^ COX6B1,^42^ MTOR,^43^ and GPX2 ^44^ in A549 cells could promote cell apoptosis after AgNPs treatment.

Interestingly, proteins participating in membrane trafficking were observed to be downregulated in both AgNP-treated A549 and A549/DDP cells. Proteins, including CUX1, CTSC, GOLGA1, and IGF2R in A549 cells and ATP1B1, ATP5F1D, and COX1 in A549/DDP cells, were downregulated upon AgNP treatment. This result suggests that even though membrane-trafficking-related proteins were less expressed, AgNPs could still exert cytotoxicity on lung adenocarcinoma cells, suggesting that the dominant way for AgNPs to enter the cells is not via membrane trafficking. Our previous findings suggested that clathrin-mediated endocytosis is the major endocytic process for AgNPs.^45,46^ This also provides a good explanation for why AgNPs could escape cisplatin resistance and induce cytotoxic effects on A549 cells regardless of their sensitivities to cisplatin.

In brief, A549 cells and A549/DDP cells similarly respond to AgNPs, unlike their responses to cisplatin, which are markedly different. These results explain why AgNPs remain effective against cisplatin-resistant A549 cells. Some studies also delineated the potency of AgNPs for overcoming multidrug-resistant cancers.^8,9^ The protein–protein interaction networks are shown in Figure 4c2,c4. Tables S3 and S4 show the altered pathways related to AgNP-induced cytotoxicity in both A549 and A549/DDP cells.

### In Vivo Assessments for Anticancer Efficacy of AgNPs in NSCLC-Bearing Mice

We established mice xenograft models with both cisplatin-sensitive and cisplatin-resistant NSCLC variants for *in vivo* studies of AgNPs (Figure 5a). We conducted immunohistochemical experiments to assess the anticancer efficacy of AgNPs. Besides, we studied the biodistribution of AgNPs in the mice xenograft models for the *in vivo* toxicity studies. In addition, healthy SD rats administered with AgNPs through tail-vein injection were used for pharmacokinetics studies.

**Figure 5:**
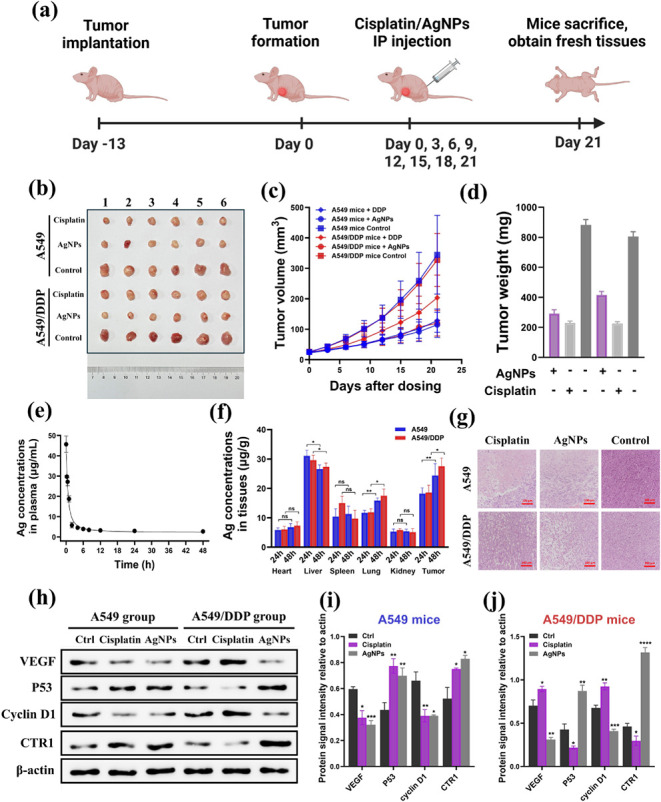
*In vivo* evaluation of AgNPs in antitumor efficacy using A549 and A549/DDP-bearing nude mice models. (a) Schematic illustration of the establishment of A549 and A549/DDP mice xenograft models for *in vivo* studies. (b) Photograph of tumors excised from the mice at day 21. (c) The average tumor volume in each group upon treatments. (d) Average tumor weight in each group at day 21. (e) Concentration–time curve of AgNPs in healthy mice blood plasma after intraperitoneal injection. (f) Biodistribution of AgNPs in organ tissues and tumors after intraperitoneal administration at 48 h. Data are the Mean ± SD of *n* = 3. *p < 0.05, **p < 0.01, ***p < 0.005, ****p < 0.001 by *t*-test. (g) Histological assessments of tumors from A549 and A549/DDP mice after treatments on day 21. (h) Representative immunoblotting analysis of tumors from A549 and A549/DDP mice after treatments at day 21, with quantitative analysis in (i) and (j). Quantitative results were obtained by the relative intensity of proteins in comparison to actin, and the plots were expressed in Mean ± SEM of *n* = 3. *p < 0.05, **p < 0.01, ***p < 0.005, ****p < 0.001 in comparison to control groups by *t*-test.

The AgNPs concentration in healthy rat plasma was monitored and measured by ICP-MS at different time points, including 0, 0.25, 0.5, 1, 2, 4, 6, 8, 12, 24, and 48 h post injection (Figure 5e). In rat plasma, AgNPs were cleared rapidly, with *t*_1/2_ in less than an hour. This result suggested that AgNPs could be translocated quickly and accumulated in tissues via circulatory systems, which is consistent with previous studies on size-dependent clearance of nanoparticles.^47^ Moreover, we examined the biodistribution of AgNPs in A549 and A549/DDP-bearing mice at 24 h and 48 h post injection. We found that AgNPs accumulated mostly in the liver and the grafted tumors, and comparatively less in the heart, kidney, spleen, and lungs. Interestingly, the accumulation of AgNPs in the liver (A549: 31.07 ± 1.90 μg/g and A549/DDP: 29.61 ± 1.68 μg/g at 24 h; A549: 26.60 ± 1.37 μg/g and A549/DDP: 27.39 ± 1.23 μg/g at 48 h) decreased with time, suggesting that detoxification might be happening in the liver. In contrast, AgNP concentrations increased with time in tumors in both models. Besides, H&E-stained tumor samples from the A549 and A549/DDP groups treated with AgNPs exhibited significant damage compared to the PBS control groups (Figure 5g). No notable morphological differences were observed in major organs—including the heart, liver, spleen, lungs, and kidneys—between the AgNP-treated and control groups, suggesting that AgNPs have minimal toxicity to most organs in mice (Figures S9 and S10). To further evaluate the *in vivo* AgNP toxicity, liver enzymatic biomarkers were examined in mice serum after 21 days of drug treatment. It is found that there were no significant changes in the activities of three classical liver enzymes induced by AgNPs in both A549 and A549/DDP mice compared to their control groups, further supporting the low *in vivo* toxicity of AgNPs (Figure S11). For the mice xenograft studies, all mice were euthanized, and their tumors were collected, weighed, and photographed after 21 days of treatment. Our results indicated that AgNPs significantly reduced tumor burdens, including size, volume, and weight, in both A549 and A549/DDP-bearing mice (Figure 5b–d). The average tumor mass was 291.98 ± 62.25 mg for the A549 group and 415.97 ± 56.85 mg for the A549/DDP group in the AgNP-treated mice, compared to 882.23 ± 88.47 mg and 805.32 ± 78.04 mg in their control groups. Furthermore, the tumor volumes in the AgNP-treated groups were notably smaller, measuring 128.16 ± 38.08 mm^3^ and 203.53 ± 74.18 mm^3^ in the A549 and A549/DDP mice, respectively, compared to 344.62 ± 129.34 mm^3^ in the A549 control group and 327.35 ± 87.23 mm^3^ in the A549/DDP control group. The body weights of mice in all groups did not show a drastic decrease, suggesting that AgNPs do not pose severe toxic effects to mice (Figure S8).

To investigate the mechanism behind the effectiveness of AgNPs in both cisplatin-sensitive and cisplatin-resistant lung adenocarcinoma, we conducted a Western blotting experiment to analyze the regulation of proteins in key altered pathways following treatment (Figure 5h). We selected four protein targets to measure, based on our findings from quantitative proteomics on the cell lines. These proteins are key proteins that regulate pathways of cytotoxic and anticancer properties in lung adenocarcinoma that are triggered by AgNPs. These proteins are: (1) VEGF that regulates angiogenesis, (2) P53 that mediates cell apoptosis, (3) Cyclin D1 that controls cell cycle progression, and (4) CTR1 that regulates the influx of metals.

First, AgNPs are known to inhibit VEGF-induced cell proliferation, thereby suppressing cell growth.^48^ Our findings indicated that AgNPs can downregulate VEGF expression in both A549 and A549/DDP mice, which is consistent with our proteomic data. Besides, we observed that P53 expression was upregulated in both cisplatin-sensitive and -resistant lung tumors. Previous studies have shown that the activation of p53-mediated pathways can inhibit cell apoptosis, which is a target of AgNPs.^49^ Furthermore, cell cycle arrest is a significant cause of cell death, and cyclins play a crucial role in regulating cell cycle progression. Our Western blot results revealed that AgNPs can reduce the levels of cyclin D1, which is often overexpressed in various cancer cells.^50^ Consequently, the reduced cyclin D1 levels may impede G1-S transitions and lead to apoptotic cell death. Notably, the CTR1 protein plays an essential role in the accumulation of AgNPs within mammalian cells, and recent studies have shown that AgNPs treatment significantly upregulates CTR1 expression.^51^ This increased accumulation of AgNPs in cells suggests a corresponding increase in AgNPs-induced toxicities. Our results demonstrated that CTR1 levels increased in lung cancer cells following AgNPs treatment, indicating that enhanced metal influx may also contribute to cell apoptosis. As a consequence, our results showed that AgNPs altered protein expression in key apoptotic pathways and subsequently induced cell death.

## Conclusion

In the present study, we explored the mechanisms of action and potential anticancer properties of AgNPs on cisplatin-sensitive and -resistant lung adenocarcinoma by comparative proteomic analysis and mice xenograft models, exposing them to AgNPs and comparing them to cisplatin. The comparison considers that AgNPs and cisplatin are believed to be cytotoxic largely due to the unique AgNPs-induced toxicity and their heavy metal content. Still, it is of practical interest to know whether AgNPs can be effective against cisplatin-resistant cancer cells and, if so, the underlying mechanism of action. Here, we found that AgNPs could induce cell death in both A549 and A549/DDP cells in a similar way by increasing cell cycle arrest, disrupting DNA damage repair, suppressing the VEGF signaling pathway, increasing ROS generation, and hampering mitochondrial function. On the other hand, A549/DDP cells are less sensitive to cisplatin because of the decrease in influx transport proteins, which explains the lower intracellular cisplatin concentration in A549/DDP cells compared to A549 cells. Aligned with *in vivo* experiments, AgNPs exhibited excellent antitumoral effects on both A549 and A549/DDP-grafted mice, regardless of their sensitivity to the chemotherapeutic drug. Figure 6 summarizes the proposed cytotoxic and anticancer mechanisms in cisplatin-sensitive and -resistant lung adenocarcinoma exposed to cisplatin and AgNPs. In summary, this study investigated the action mechanisms of AgNPs in both cisplatin-sensitive and -resistant lung adenocarcinoma using quantitative proteomics and a mice xenograft model, which offers a systematic and holistic view into the cellular responses that should help assess the potential of AgNPs as anticancer agents.

**Figure 6:**
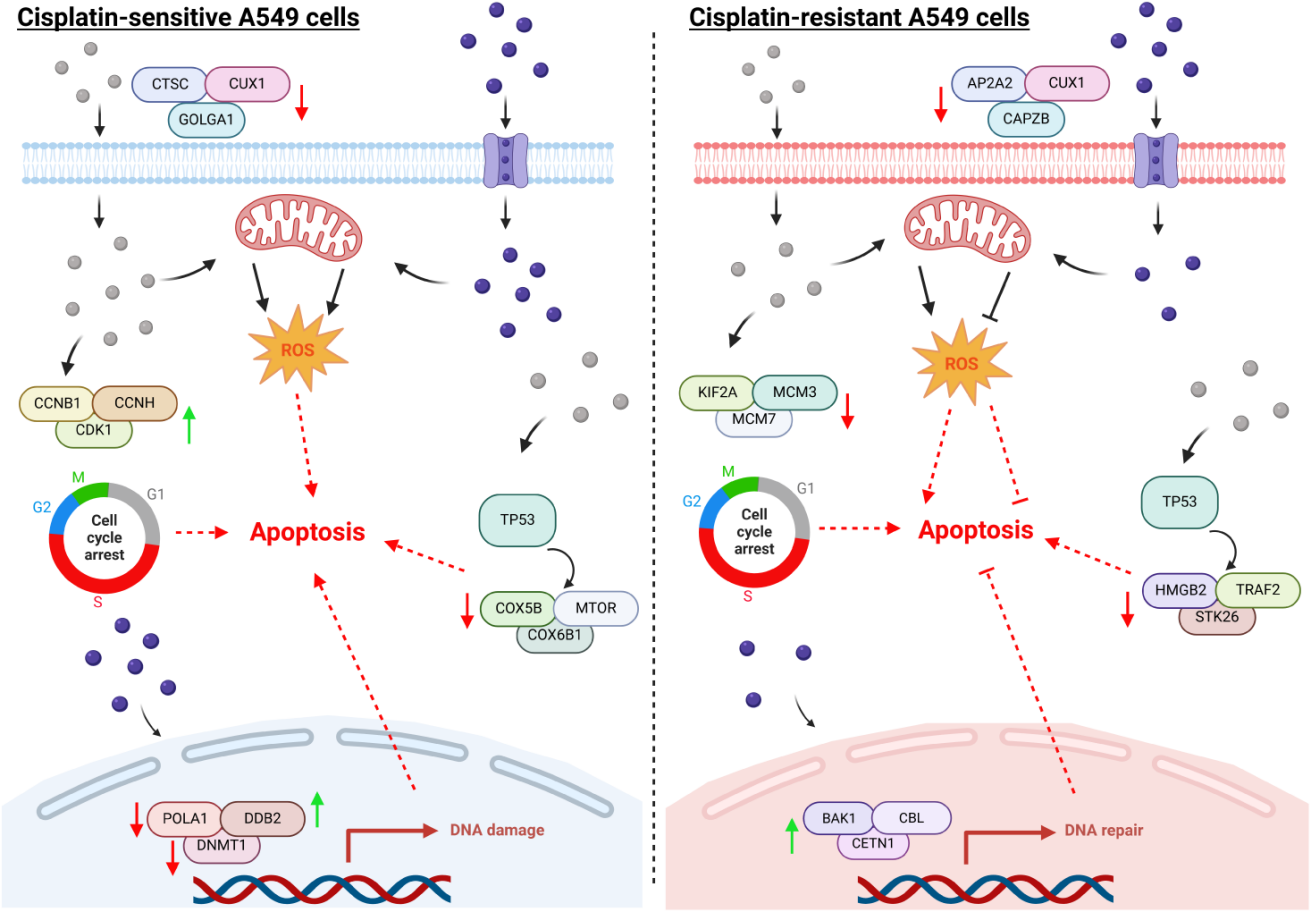
Schematic diagram of the proposed cytotoxic and anticancer mechanisms induced by cisplatin (spheres in purple) and AgNPs (spheres in silver) in A549 and A549/DDP cells, respectively.

## Experimental Section

### AgNPs Synthesis and Characterization

Unless otherwise specified, chemicals with a purity equal to or greater than 99% were obtained from Sigma-Aldrich (Merck). To synthesize AgNPs, we used the methods we previously reported.^52^ In detail, a mixture containing 0.25 mM sodium citrate and 0.25 mM AgNO_3_ was prepared, and then 6.0 mL of 5 mM NaBH_4_ was added dropwise to the mixture. A yellowish solution was obtained after vigorous mixing and stirring for 30 min. To purify the solution, centrifugal ultrafiltration was employed. The purified AgNPs were subsequently resuspended in 2 mL of deionized water. The concentration of AgNPs was determined using inductively coupled plasma mass spectrometry (ICP-MS, NexION 300X, PerkinElmer, USA) following nitric acid digestion.^53^ The sizes of AgNPs were characterized using transmission electron microscopy (TEM) (JEOL 2010 TEM). Additionally, the hydrodynamic diameter and zeta potential of AgNPs were analyzed using dynamic light scattering (DLS) with a Malvern Instrument Zetasizer Nano Series (Malvern Instruments, Westborough, MA, USA) equipped with a He–Ne laser (λ = 633 nm, max 5 mW) and operated at a scattering angle of 173°. The UV–vis spectra of AgNPs were recorded using a Varian Cary 50 Conc UV–visible spectrophotometer, and the Fourier-transform infrared spectroscopy (FTIR) spectra of AgNPs were obtained using a PerkinElmer Spectrum 65 series FTIR spectrophotometer.

### Human Lung Adenocarcinoma Cell Lines Culture and Cell Viability Test

The human lung adenocarcinoma cells were obtained from the Shanghai Cell Bank, including cisplatin-resistant (A549/DDP) cells and their progenitor A549 cells. The cells were cultured in RPMI 1640 medium supplemented with stable glutamine, 10% fetal bovine serum (FBS), and 1% penicillin-streptomycin. The cultures were maintained at 37 °C in a water-jacketed CO_2_ incubator with a 5% CO_2_ atmosphere. Cell passages ranging from 3 to 6 were used for the experiment.

To assess the cell viability, an MTT assay was performed. A549/DDP and A549 cells were seeded in a 96-well plate and incubated at 37 °C with 5% CO_2_ for 24 h to reach a cell density of 1.0 × 10^4^ cells/mL. After overnight cell attachment, cisplatin-sensitive and -resistant lung adenocarcinoma cells were treated with different concentrations of cisplatin or AgNPs in a complete culture medium for 24 h. The concentrations used for exposure were 0, 1.25, 2.5, 5, 10, 15, and 20 μM for cisplatin and 0, 2.5, 5, 7.5, 10, 12.5, and 15 μg/mL for AgNPs. After cell exposure, MTT (3-(4,5-dimethylthiazol-2-yl)-2,5-diphenyltetrazolium bromide) reagent was added to each well at a final concentration of 0.5 mg/mL. The plate was kept in the dark to prevent light exposure and incubated at 37 °C with 5% CO_2_ for 3 h. Subsequently, 100 μL of dimethyl sulfoxide (DMSO) was added to all wells, and the plate was gently shaken to ensure complete dissolution of the formazan crystals. Finally, the plates were measured at an absorption wavelength of 570 nm using a microplate reader (Varioskan LUX multimode, Thermo Fisher, USA). The results were analyzed by normalizing the percentage of cell viability.

### Intracellular Uptake and Localization of AgNPs and Cisplatin in Lung Cancer Cells

The intracellular concentrations of AgNPs (silver, Ag) and cisplatin (platinum, Pt) were quantified using inductively coupled plasma-mass spectrometry (ICP-MS) with the NexION 300X instrument (PerkinElmer, USA). Cells were seeded onto six-well plates and allowed to grow for 24 h. Subsequently, the cells were exposed to a concentration of 2.5 μg/mL AgNPs or 10 μM cisplatin for 24 h. After exposure, the cells were washed three times with 1× PBS and trypsinized. The cells were then harvested by centrifuging at 5000 × *g* for 5 min. The obtained cell pellets were digested with concentrated nitric acid overnight at 85 °C and stored at −20 °C until analysis by ICP-MS.

### Measurement of Intracellular Reactive Oxygen Species (ROS)

The intracellular levels of ROS were assessed by using CM-H_2_DCFDA dye (Thermo Fisher Scientific) and measured with a UV–vis spectrometer. For this experiment, A549/DDP and A549 cells were seeded in a 6-well plate and incubated at 37 °C in a water-jacketed incubator with 5% CO_2_ for 24 h. Subsequently, the cells were exposed to AgNPs (2.5 μg/mL) or cisplatin (10 μM) for an additional 4 h. After that, the cells were incubated with CM-H_2_DCFDA at 37 °C for 30 min. Following two washes with 1× PBS, the cells were collected by using trypsin and measured at an excitation wavelength of 488 nm and an emission wavelength of 530 nm by using a UV spectrometer.

### Flow Cytometric Analysis of Cell Apoptosis

Cellular apoptosis was evaluated using a flow cytometer (BD IIIu, USA) and an Annexin V-FITC Apoptosis Detection Kit (Sigma-Aldrich, USA). A549 and A549/DDP cells were seeded in a 6-well culture plate and allowed to reach a cell density of 1 × 10^5^ cells/mL at 37 °C with 5% CO_2_ supplementation. For the treated groups, the cells were subsequently exposed to a concentration of 2.5 μg/mL AgNPs or 10 μM cisplatin for 24 h. Following exposure, the trypsinized cells were harvested and stained with Annexin V-FITC/PI for 30 min before detection of apoptotic cells.

### Flow Cytometric Analysis of Internalization Pathways

To study the internalization pathways of AgNPs, cells were incubated for 30 min with endocytosis inhibitors—sodium azide (NaN_3_, 0.25 mM), monodansylcadaverine (MDC, 0.2 mM), and filipin complex (FC, 5 μM)—before AgNPs exposure to block the different endocytic routes and evaluate the impact of endocytosis on AgNPs internalization by comparing with the amount of AgNPs internalized without the addition of inhibitors. A time period of 24 h was chosen to assess the endocytosis of the cells, and flow cytometry was used for the intracellular determination of AIE-NPs after the treatment with different inhibitors.

### Confocal Laser Scanning Microscopic (CLSM) Imaging of Live Cells

For the biodistribution studies, the lysosomes were labeled using a lysosome tracker (LysoTracker Green DND-26, Invitrogen, USA), and the mitochondria were stained with a mitochondria tracker (MitoTracker Red FM, Thermo Fisher, USA), and the AgNPs were visualized in reflection mode. In addition, mitochondria morphology and interconnectivity were examined by staining the mitochondria with a mitochondria tracker (MitoTracker Deep Red DND-99, Invitrogen, USA).

Specifically, human lung adenocarcinoma cells were treated with a concentration of 2.5 μg/mL AgNPs or 10 μM cisplatin under standard cell culture conditions for 24 h. The RPMI 1640 medium was then removed, and the cells were washed with 1× PBS thrice. Fresh RPMI 1640 medium was added together with the respective stains. After a 30-min incubation at 37 °C, the human lung adenocarcinoma cells were washed twice with 1× PBS and imaged using a confocal microscope equipped with ZEN Blue software (LSM980, Carl Zeiss).

For imaging AgNPs, the reflection mode was employed using a HeNe laser and a femtosecond pulse with a pinhole of 0.3 Å. The confocal fluorescence imaging settings were configured as follows: DAPI channel, λ_ex_ = 405 nm and λ_em_ = 420–500 nm; lysosome tracker channel, λ_ex_ = 633 nm and λ_em_ = 650–710 nm; mitochondria tracker channel, λ_ex_ = 633 nm and λ_em_ = 645–730 nm; AgNPs in reflection mode, λ_ex_ = 488 nm and λ_em_ = 483–493 nm.

### Cell Mito Stress Test by Seahorse XFp Analyzer

The oxygen consumption rate (OCR) was assessed using an XFp extracellular analyzer (Seahorse Bioscience, USA). The measurements were conducted at 37 °C supplied with 5% CO_2_. Briefly, A549 and A549/DDP cells were seeded into a Seahorse XFp cell culture microplate at a density of 1.0 × 10^4^ cells/well and allowed to adhere overnight at 37 °C with 5% CO_2_ supplementation. The sensor cartridge was moisturized in a CO_2_-free incubator at 37 °C overnight. Subsequently, 10 μM of cisplatin or 2.5 μg/mL of AgNPs were added to the corresponding wells and incubated for an additional 24 h.

Assay media and cells were prepared following the protocols provided by the manufacturer, and a Mito Stress Test Kit was utilized.^54^ Real-time OCR monitoring was conducted by sequentially injecting 1.0 μM oligomycin, 1.0 μM carbonyl cyanide 4-(trifluoromethoxy)­phenylhydrazone (FCCP), and 0.5 μM Antimycin A/Rotenone. The obtained raw data were analyzed using Wave software, and the Student’s *t*-test was employed to compare the average values between different conditions.

### Sample Preparation for Proteomic Studies

For proteomics studies, A549 and A549/DDP cells were grown to a confluency of 80% on 100 mm TC-treated culture dishes at 37 °C supplied with 5% CO_2_. The cells were treated with 2.5 μg/mL AgNPs or 10 μM cisplatin for 24 h. After exposure to AgNPs or cisplatin, the culture medium was removed, and the cells were washed three times with 1× PBS. Subsequently, the cells were trypsinized and collected. The cells were then resuspended in 1× PBS and harvested by centrifugation at 5000 × *g*. They were then washed three times with cold 1× PBS.

The resulting cell pellets were resuspended in 300 μL of lysis buffer containing 8 M urea and flash-frozen in liquid nitrogen for 1 min. Cell rupture was achieved by sonication on ice for 15 min. The cell lysate was centrifuged at 16,000 × *g* for 15 min at 4 °C, and the obtained supernatant was mixed with four volumes of ice-cold acetone and stored at −20 °C overnight. The precipitated proteins were pelleted by centrifugation at 16,000 × *g* for 10 min at 4 °C and then washed with 80% acetone. The remaining solvents were left to evaporate, and the proteins were reconstituted in a buffer containing 4 M urea and 25 mM ammonium bicarbonate. The protein concentrations in the samples were determined by using the Bradford protein assay (Bio-Rad Protein Assay Kit) with bovine serum albumin (BSA) as the protein standard.

The protein samples were first incubated with 10 mM dithiothreitol (DTT) at 37 °C for 1 h. Subsequently, a final concentration of 20 mM iodoacetamide (IAA) was added, and the treated samples were incubated in the dark at room temperature for 30 min. The reaction was quenched by adding a final concentration of 10 mM DTT solution, and the samples were diluted with a 25 mM ammonium bicarbonate solution. Reduced and alkylated proteins were digested using sequencing-grade modified trypsin (1:50 w/w, Promega, Madison, WI) at 37 °C for 16 h. The solution was acidified with 10% formic acid to a final concentration of 0.1% (v/v) and desalted and purified using a ZipTipC18 (C18-resin packed pipette tip). Finally, the purified peptides were dried using a SpeedVac (Eppendorf, Hamburg, Germany) and stored at −20 °C until LC-MS/MS analysis.

### High-Performance Nanoflow Liquid Chromatography

Bottom-up proteomics analysis was conducted using an ultraperformance liquid chromatography coupled to a tandem mass spectrometer (UPLC-MS/MS) system. Specifically, the Bruker nanoElute ultrahigh-performance liquid chromatography (Bruker Daltonics, Bremen, Germany) was coupled to a trapped ion mobility-quadrupole time-of-flight mass spectrometer (TimsTOF Pro, Bruker Daltonics, Bremen, Germany) and operated using the online Parallel Accumulation Serial Fragmentation (PASEF) acquisition method.

For the LC-MS/MS analysis, approximately 200 ng of protein digest was reconstituted and loaded onto the UHPLC system. Separation was achieved using a C18 analytical column (IonOpticks 25 cm Aurora Series Emitter column with Captive Spray Insert, 120 Å pore size, 1.6 mm particle size, 250 mm × 75 μm ID). The chromatographic run employed a 30-min step gradient at a flow rate of 300 μL/min, with the column temperature maintained at 50 °C.

The mobile phases used were as follows: mobile phase A, consisting of 0.1% formic acid in water, and mobile phase B, composed of 0.1% formic acid in acetonitrile. The chromatography gradient started with 2% solvent B, increased linearly to 10% over 4 min, held at 10–32% for 60 min, increased to 32–60% over 6 min, and further increased to 60–80% in 2 min, followed by 25 min at 100% solvent B. Finally, 2% solvent B was maintained for column reequilibration prior to the next sample injection.

### Tandem Mass Spectrometry

Tandem mass spectrometry was conducted using the Bruker TimsTOF Pro mass spectrometer, as described in refs. 55 and 56. The parameters for the experiment were set as follows: the ramp time and accumulation time were both set to 100 ms, and mass spectra were recorded in the positive electrospray mode over a range of *m*/*z* 100–1700. The ion mobility was scanned from 0.85 to 1.30 Vs/cm^2^.

During the analysis, a full TIMS-MS scan was acquired along with four parallel accumulation-serial fragmentation (PASEF) MS/MS. The cycle time for these scans was 0.53 s. To calibrate the TIMS dimension, three ions from the Agilent ESI LC/MS tuning mix [*m*/*z*, 1/K_0_: (622.0289, 0.9848 Vs/cm^2^), (922.0097, 1.1895 Vs/cm^2^), (1221.9906, 1.3820 Vs/cm^2^)] were selected in positive mode.

### Proteomics Data Analysis

The raw data obtained from the timsTOF Pro mass spectrometer were initially converted into MGF files and then further transformed into mzXML files using ProteoWizard’s MSConvert program (version 3.0.11676, 64-bit), as described previously.^57^ Subsequently, the mzXML files were subjected to a search analysis using the open-source tool Comet (version 2019.04 rev.0)^58^ within the Trans-Proteomics Pipeline (version 5.1.0).^59^ The search was performed against the human protein sequence database obtained from UniProt (Swiss-Prot). To estimate the peptides identified with a controlled false discovery rate (FDR), decoy sequences were created by shuffling amino acid sequences between tryptic cleavage sites and then incorporating them into the database.

The Comet search parameters were set as follows: 40 ppm peptide mass tolerance, monoisotopic mass type, fully digested enzyme termini, 0.05 amu fragment bin tolerance, 0 amu fragment bin offset, and variable modification for oxidized methionine and fixed modification for carbamidomethylated cysteine. The search results generated by Comet were subsequently processed using PeptideProphet,^60^ iProphet, and ProteinProphet, which are tools integrated into the Trans-Proteomics Pipeline (TPP) and operate in the decoy-assisted nonparametric mode. Each mzXML run was analyzed independently. Protein identifications were filtered using a false discovery rate (FDR) threshold of 0.01.

The label-free protein quantification method employed in this study utilized NSAF values, as described previously.^61^ The search results were obtained by combining the two technical replicates for each biological replicate and considering proteins identified in at least two-thirds of the biological replicates. To identify differentially expressed proteins (DEPs) between the groups treated with AgNPs and the control group, the Student’s *t*-test was employed to evaluate the difference of means of the NSAF values between groups. DEPs were defined as proteins showing fold changes higher than 1.5-fold or lower than −1.5-fold, with a *p*-value below 0.05.

### Bioinformatics Analysis

The protein–protein interactions (PPI) of the significantly regulated proteins were predicted using STRING (version 11.0) with high confidence (i.e., interaction score = 0.900), as previously described.^62^ Gene annotation and enrichment analysis were performed using Metascape.^63^ For gene ontology (GO) and pathway analysis, the DAVID (Database for Annotation, Visualization and Integrated Discovery) tool (version 6.8)^64^ was utilized. All networks were visualized using Cytoscape software (version 3.8.1), as previously outlined.^65^

### Mice Xenograft Model

Four- to six-week-old male BALB/c nu/nu nude mice, weighted ∼18–25 g, were maintained in pathogen-free conditions at 22 °C ± 2 °C, with 70% relative humidity and under a 12-h light/dark cycle. A549/DDP or A549 cells (1 × 10^7^/0.2 mL PBS) were injected subcutaneously into the left flank of the nude mice for tumor formation. Here, we will generate two types of mice: A549 tumor-bearing mice and A549/DDP tumor-bearing mice. On day 13 of post-tumor implantation, the mice were randomized into four groups (each group consisting of six biological replicates, *N* = 6) according to their tumor volume so that all groups had a similar starting mean tumor volume.

Cisplatin (3 mg/kg) was administered 2–3 times per week by intraperitoneal (IP) injection, while the AgNPs (5 mg/kg) were also administered via intraperitoneal (IP) injection 2–3 times per week. The body weight of the mice was recorded every 2–3 days. On day 21 after treatments, the mice were sacrificed, and the tumor weights were recorded. Organs were also excised for the respective in vivo experiments. All animal experiments were performed according to the National Institutes of Health Guide for the Care and Use of Laboratory Animals. The protocol was approved by the Institutional Animal Care and Use Committee of the SHRM.

### Pharmacokinetics and Biodistribution

The A549 and A549/DDP mice models were generated (as mentioned in the mice xenograft model part) for the biodistribution studies. The mice were then randomly grouped with a similar tumor volume that reached approximately 100 mm^3^, and AgNPs were administered intraperitoneally. At 24 and 48 h postinjection, the mice were sacrificed, and the tumors, heart, liver, spleen, lungs, and kidneys were collected, and the concentration of AgNPs in the samples was digested and subsequently measured by ICP-MS (EXPEC 7910, Focused Photonics Inc., Hangzhou, China).

For the pharmacokinetic studies, SD rats were used. Rats were randomly grouped and administered intravenously with AgNPs (5 mg/kg). At 0, 0.25, 0.5, 1, 2, 4, 6, 8, 12, 24, and 48 h post injection, ∼0.5 mL of blood per rat was taken from the tail vein, and the plasma was harvested. The plasma samples were digested, and the concentrations of AgNPs were measured by ICP-MS.

### Histological Staining

The tumors and organs obtained as above-mentioned were fixed in 10% formalin for 48 h. Following fixation, the tissues were embedded in paraffin blocks, then sliced into thin sections (approximately 5 μm thick) and mounted on glass slides. After washing the slides, hematoxylin and eosin (H&E) staining was performed on the tissue sections. Histological examination of the tumors and major organs was performed using a bright-field microscope (Eclipse Ni, Nikon Corporation, Tokyo, Japan) at a magnification of 40×.

### Western Blot Analysis

To investigate the mechanistic approach behind the anticancer potentials of AgNPs, the Western blot analysis was carried out in A549 and A549/DDP-bearing mice tumors.^66,67^ The protein expressions of VEGF, p53, cyclin D1, and CTR1 (with β-actin as the normalization control) were investigated in tissue lysates from both A549 and A549/DDP-bearing mice tumors. Total protein was extracted using a whole protein extraction kit (Sangon Biotech, Shanghai, China) following the manufacturer’s guidelines. A 9% sodium lauryl sulfate-polyacrylamide gel (SDS-PAGE) was employed to separate the target protein fragment, which was then transferred to a poly­(vinylidene fluoride) (PVDF) membrane. The membrane was blocked with TBST containing 5% skimmed milk for 30 min at room temperature, followed by incubation with a specific primary antibody overnight at 4 °C. Subsequently, the PVDF membranes were treated with horseradish peroxidase (HRP)-conjugated secondary antibodies (Sangon Biotech, Shanghai, China) at room temperature, and the blots were developed using a chemiluminescence visualization substrate system.

### Liver Enzymatic Tests

To further test the *in vivo* nanotoxicity of AgNPs, liver enzymatic assays were carried out using the serum of A549 and A549/DDP-bearing mice. We selected three typical liver biomarkers, including alanine transaminase (ALT), aspartate transaminase (AST), and alkaline phosphatase (ALP), to assess the *in vivo* AgNPs toxicity. We performed all the biochemical assays using enzymatic detection kits (Beyotime Biotechnology, P2722 and P3022S) and detected them on a Varian Cary 50 Conc UV–visible spectrophotometer. We followed the instructions from the manual provided and established methods for ALT,^68^ AST,^68,69^ and ALP.^70^

## Supplementary Material



## Data Availability

The mass spectrometry proteomics data have been deposited to the ProteomeXchange Consortium via the PRIDE partner repository with the dataset identifier PXD064272.

## Notes

Dissolution kinetics of 2.5 μg/mL of AgNPs in RMPI 1640 medium and in DI water (Figure S1); aggregation kinetics of AgNPs (2.5 μg/mL) in RMPI1 640 medium and in DI water (Figure S2); bioaccumulation of AgNPs and Ag^+^ in A549 and A549/DDP cells after 24 h exposure (Figure S3); impact of different endocytosis inhibitors (NaN_3_, MDC, and FC) on the bioaccumulation of AgNPs (Figure S4); Pearson correlation of the normalized spectral abundance factor (NSAF) (Figure S5); GO enrichment bar plots of the differentially expressed proteins (DEPs) (Figure S6); bar charts of the differentially expressed proteins (DEPs) (Figure S7); the body weight was checked every 3 days over a period of 21 days (Figure S8); representative H&E images of the tumor and other tissue slices from A549-bearing mice stained with H&E (Figure S9); representative H&E images of the tumor and other tissue slices from A549/DDP-bearing mice stained with H&E (Figure S10); measurement of activities of ALT, AST, and ALP in the serum after 21 days’ AgNPs exposure in A549 and A549/DDP-bearing mice (Figure S11); significantly perturbed pathways in A549 cells treated with cisplatin causing cytotoxicity (Table S1); significantly perturbed pathways in A549/DDP cells treated with cisplatin causing cytotoxicity (Table S2); significantly perturbed pathways in A549 cells treated with AgNPs causing cytotoxicity (Table S3); significantly perturbed pathways in A549/DDP cells treated with AgNPs causing cytotoxicity (Table S4) (PDF)
