# Supplementary material for: Exploring the Potentials of Silver Nanoparticles in Overcoming Cisplatin Resistance in Lung Adenocarcinoma: Insights from Proteomic and Xenograft Mice Studies
Source: ACS Nano. 2025 Sep 26;19(39):34708–23. doi: 10.1021/acsnano.5c09056 (PMC12509312; doi:10.1021/acsnano.5c09056)
Supplement: Supplementary file 1 [file nn5c09056_si_001.pdf]

Supporting information

Exploring the Potentials of Silver Nanoparticles in Overcoming Cisplatin Resistance in Lung Adenocarcinoma: Insights from Proteomic and Xenograft Mice Studies

Tin Yan Wong[a], Yan Wang[b], Kenneth Kin Leung Kwan[c], Yanrong Pan[a], Alan Ka Lun Lai[a], Sike Chen[b], Yao Xiao[d], Kun Zhou[e], Long Wu[f], Sitong Huo[b], Neng Yan[b*], Henry Lam[a*]

[a] *Department of Chemical and Biological Engineering, The Hong Kong University of Science & Technology, Clear Water Bay, Kowloon, 999077, Hong Kong, China.*

[b] *School of Environmental Studies, China University of Geosciences, 430074, Wuhan, China.*

[c] *Department of Pathology, The University of Hong Kong, 999077, Hong Kong, China.*

[d] *Department of Ocean Science, The Hong Kong University of Science and Technology, 999077, Hong Kong, China.*

[e] *Department of Bacteriology, University of Wisconsin–Madison, 53706, Madison, WI, USA.*

[f] *St. Jude Children's Research Hospital, 38105, Memphis, TN, USA*

*Corresponding author, Email: yanneng@cug.edu.cn & kehlam@ust.hk

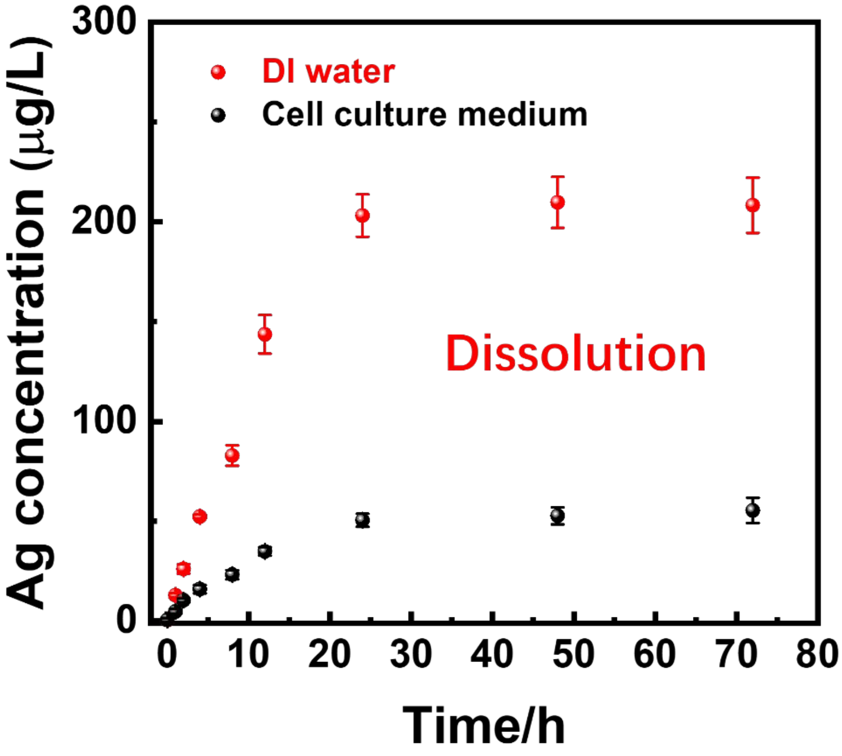

Supp Figure 1. Dissolution kinetics of 2.5 μg/mL of AgNPs in RMPI 1640 medium and in DI water.

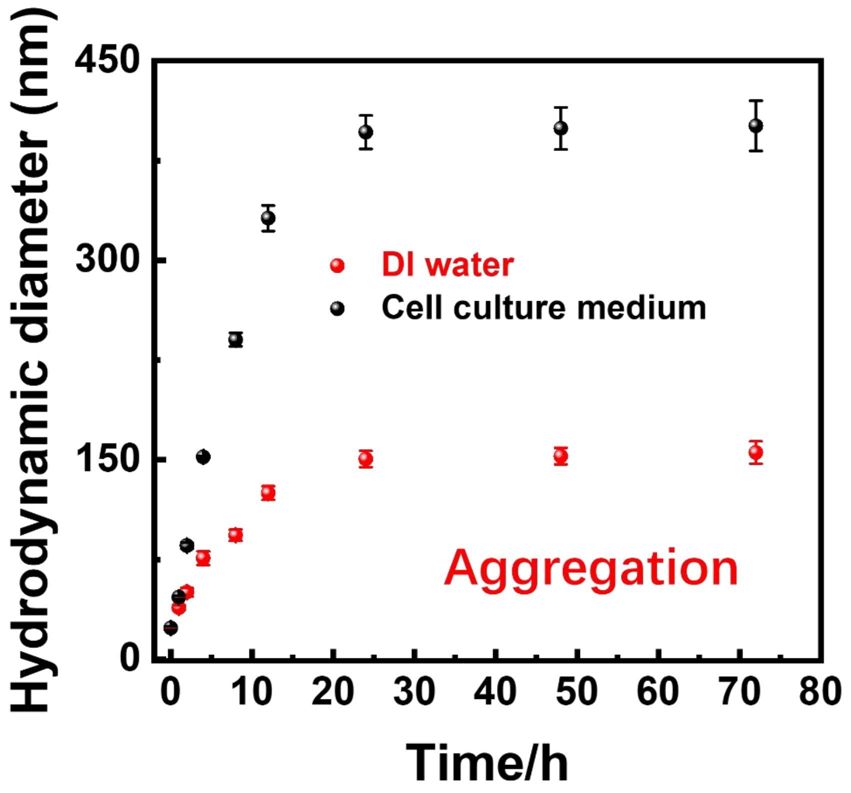

Supp Figure 2. Aggregation kinetics of AgNPs (2.5 μg/mL) in RMPI 1640 medium and in DI water.

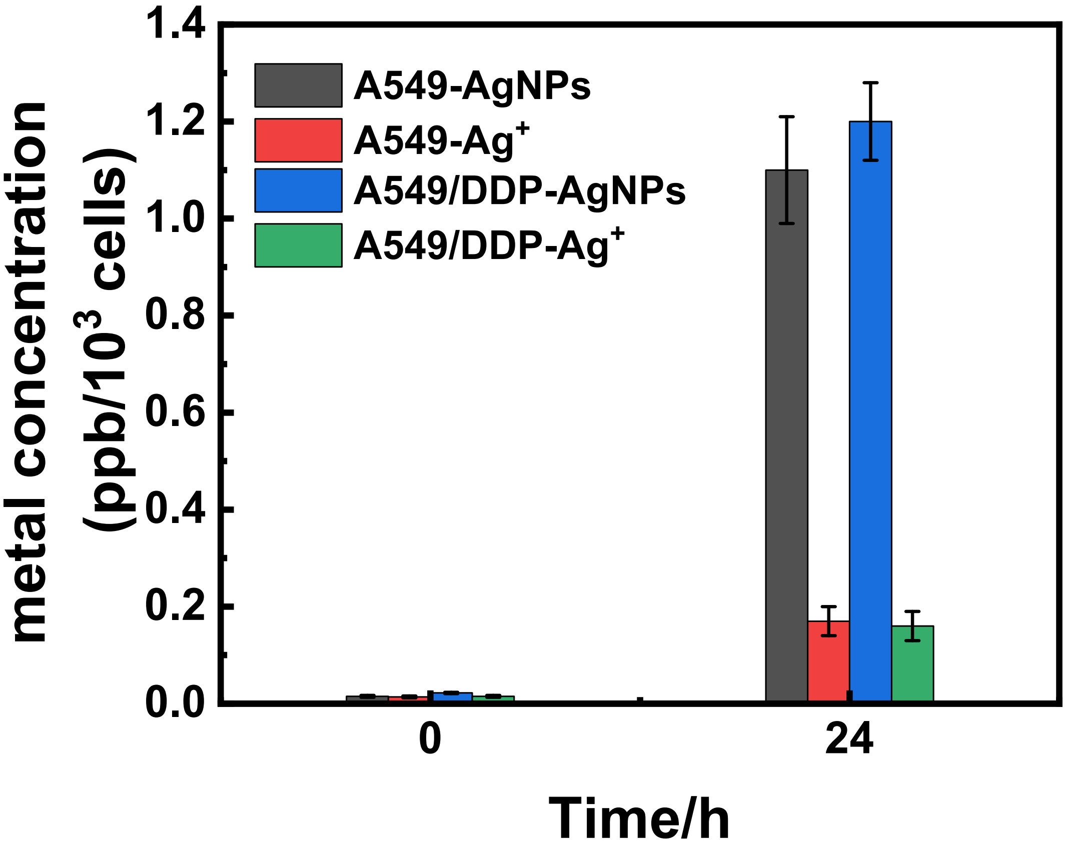

Supp Figure 3. Bioaccumulation of AgNPs and Ag^+ in A549 and A549/DDP cells after 24 hours exposure.

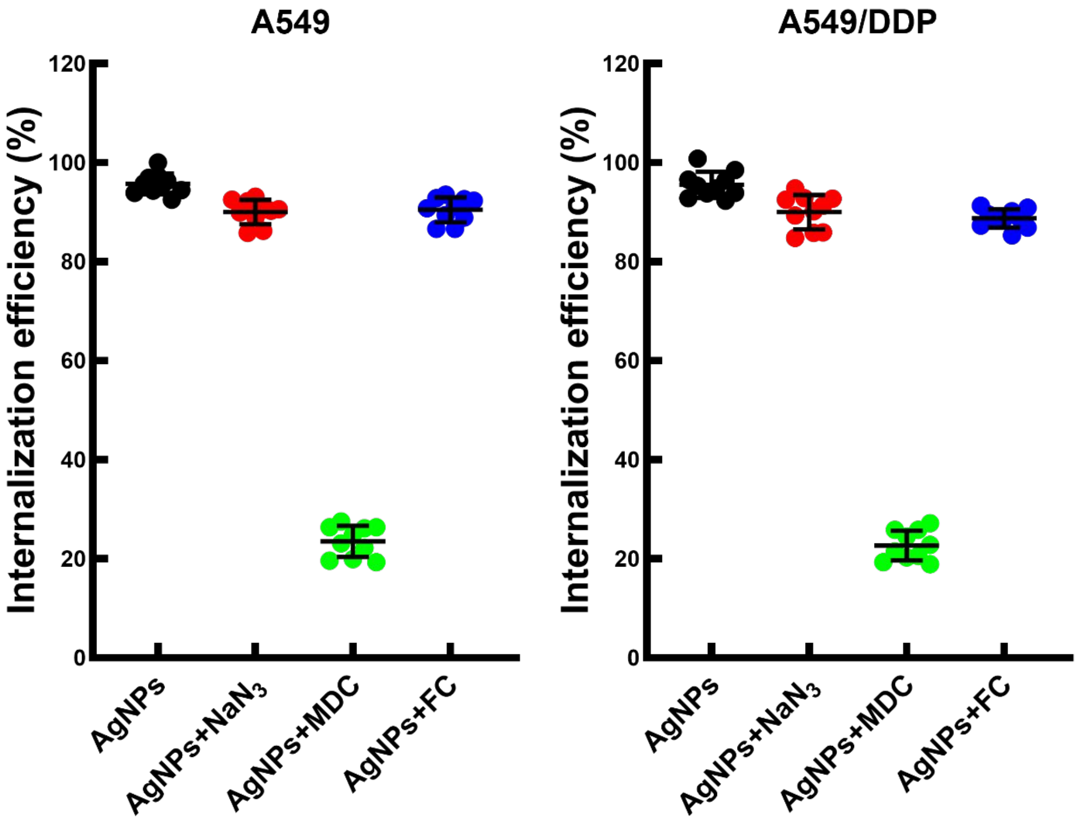

Supp Figure 4. Impact of different endocytosis inhibitors (NaN_3, MDC and FC) on the bioaccumulation of AgNPs. Human lung adenocarcinoma cells (A549 and A549/DDP cells) were first incubated with sodium azide (NaN_3, 0.25mM, a non-selective inhibitor of ATP-dependent endocytosis in general), monodansylcadaverine (MDC, 0.2 mM, an inhibitor of the clathrin-dependent endocytosis pathway) or filipin complex (FC, 0.07 mM, an inhibitor of the caveolin-dependent endocytosis pathway) before the exposure of 2.5 μg/mL AgNPs.

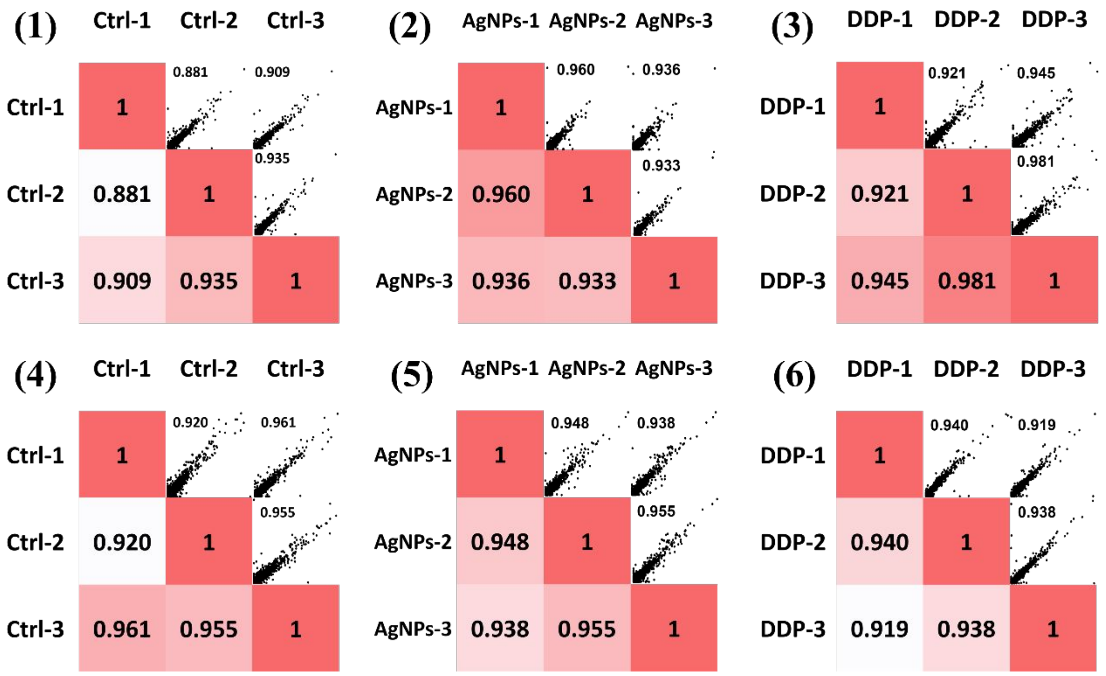

Supp Figure 5. Pearson correlation of the Normalized Spectral Abundance Factor (NSAF) between biological replicates of (1) untreated A549 cells, (2) 2.5 μg/mL AgNPs-treated A549 cells, (3) 10 μM cisplatin-treated A549/DDP cells, (5) 2.5 μg/mL AgNPs-treated A549/DDP cells, (6) 10 cisplatin-treated A549/DDP cells. Three replicates with three combinations are in the colour-scale plot, and every two replicates are compared to evaluate the reproducibility of mass spectrometry data.

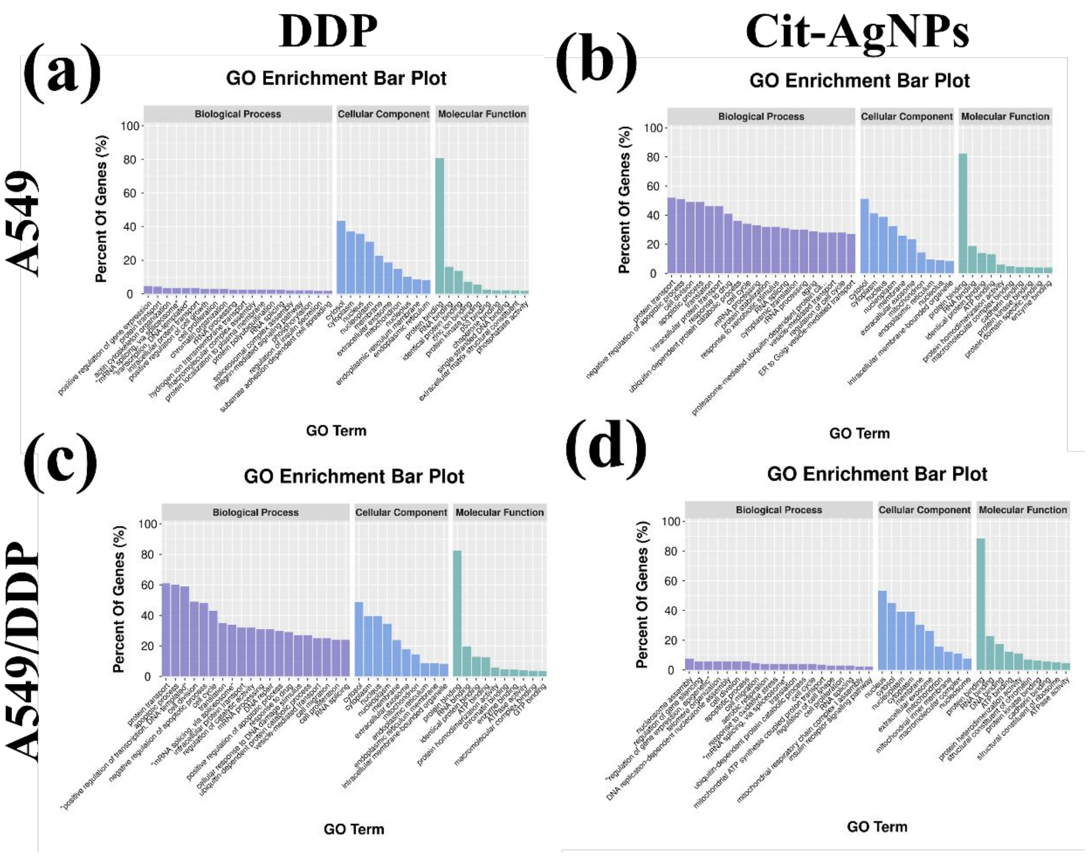

Supp Figure 6. GO enrichment bar plots of the differentially expressed proteins (DEPs) of **(a)** 2.5 µg/mL AgNPs and **(b)** 10 µM cisplatin-treated A549 cells compared to untreated A549 cells, and **(c)** 2.5 µg/mL AgNPs- and **(d)** 10 µM cisplatin-treated A549/DDP cells compared to untreated A549/DDP cells. Figures were plotted and categorised into cellular component (CC), biological processes (BP), and molecular function (MF).

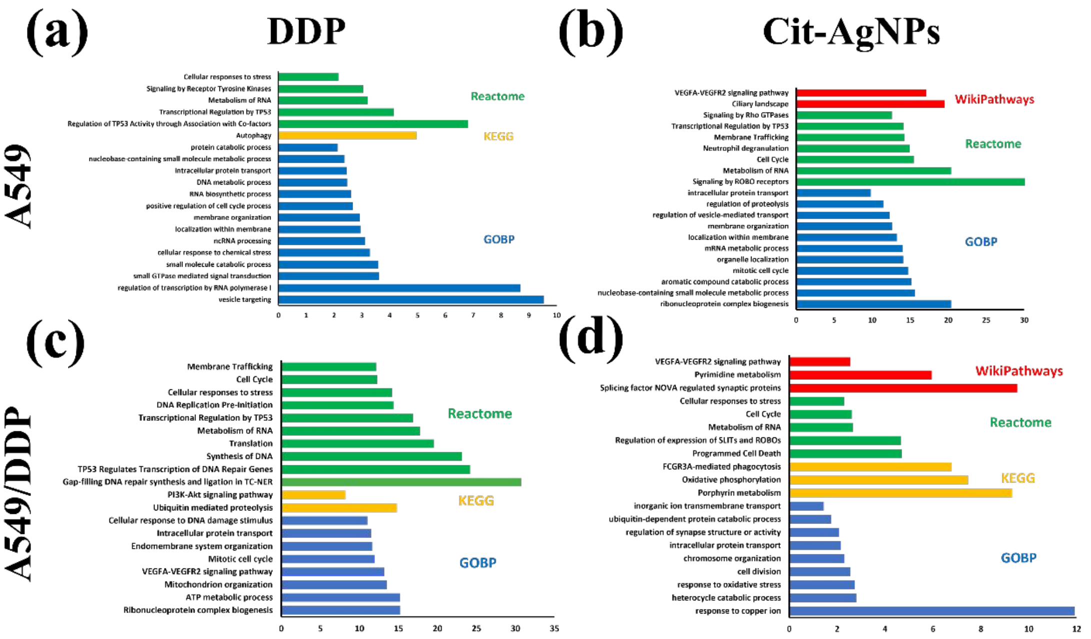

Supp Figure 7. Bar charts of the differentially expressed proteins (DEPs) of (a) 2.5 μg/mL AgNPs-treated A549 cells compared to untreated A549 cells, (b) 10 μM cisplatin-treated A549 cells compared to untreated A549 cells, (c) 2.5 μg/mL AgNPs-treated A549/DPP cells compared to untreated A549/DDP cells (d) 10 μM cisplatin-treated A549/DDP cells compared to untreated A549/DDP cells were plotted with top 20 statistically most enriched terms, including GOBP, Reactome, KEGG, and WikiPathways. Accumulative hypergeometric p-values and enrichment factors were calculated and used for filtering.

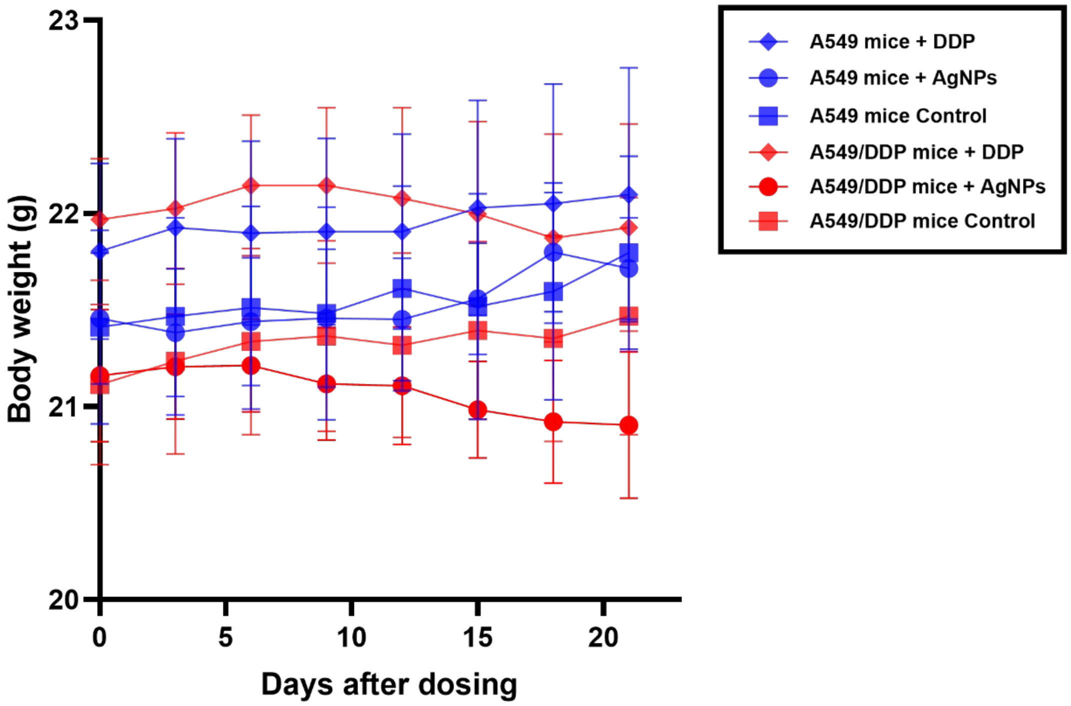

Supp Figure 8. The body weight was checked every 3 days over a period of 21 days.

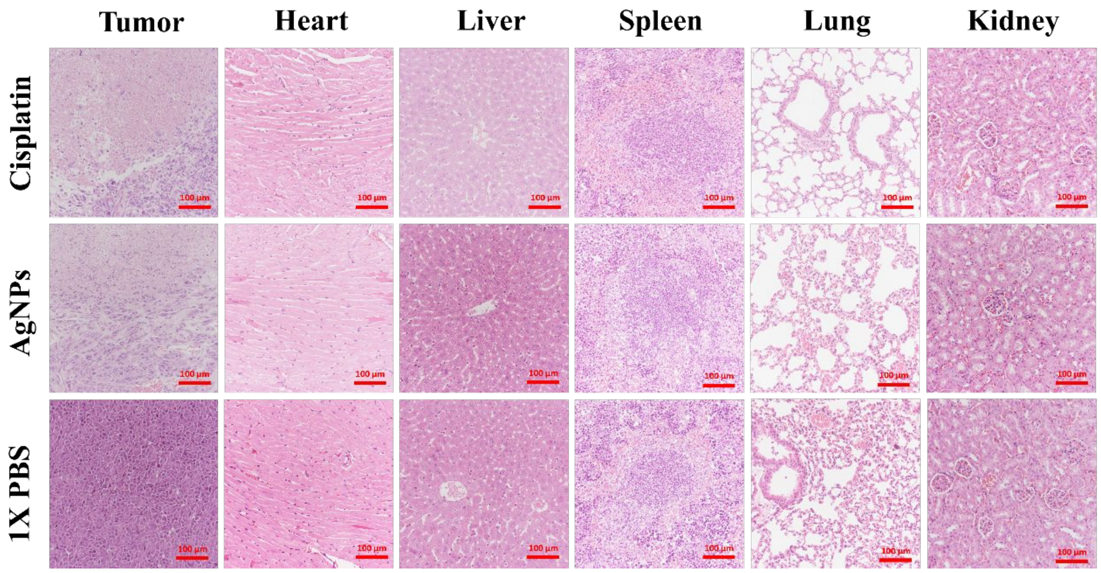

Supp Figure 9. Representative H&E images of the tumor and other tissue slices from A549-bearing mice stained with H&E.

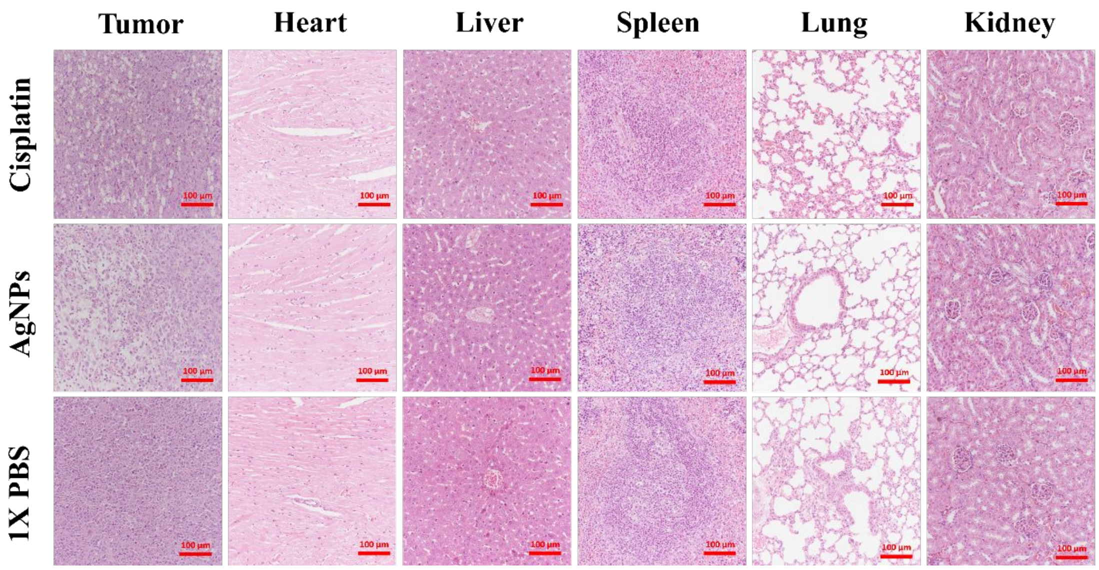

Supp Figure 10. Representative H&E images of the tumor and other tissue slices from A549/DDP-bearing mice stained with H&E.

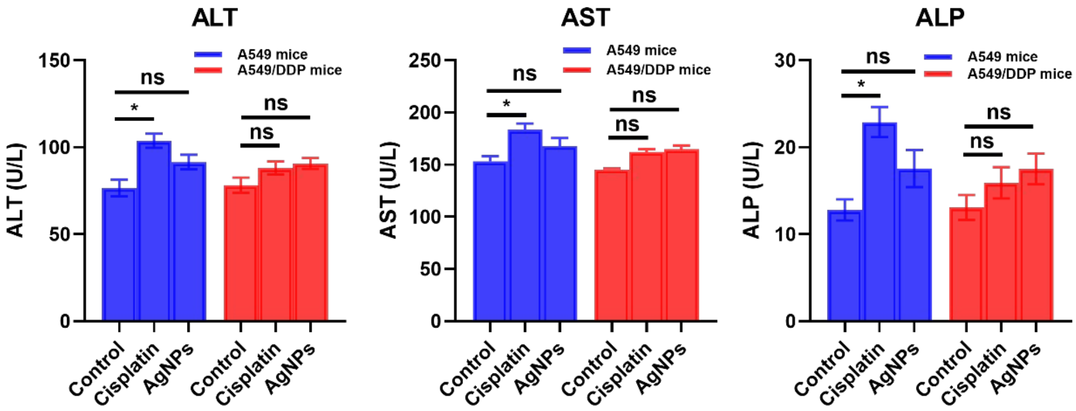

Supp Figure 11. Measurement of activities of ALT, AST, and ALP in the serum after 21 days' AgNPs exposure in A549 and A549/DDP-bearing mice. The data are presented as mean ± SD of n = 5. *$p < 0.05$ in comparison to control.

Supp Table 1. Significantly perturbed pathways in A549 cells treated with cisplatin causing cytotoxicity.

Pathway	Gene Name	Protein Name	p-value	Log2FC
Regulation by TP53	AKT1	AKT Serine/threonine Kinase 1	0.015	-0.93
	FANCD2	FA Complementation Group D2	1.99E-06	-6.13
	MTOR	Mechanistic Target of Rapamycin Kinase	0.0012	-1.52
	PRKAB1	Protein Kinase AMP-activated Non-catalytic Subunit Beta 1	0.0087	2.51
	TP53BP2	Tumor Protein p53 Binding Protein 2	0.026	1.55
DNA damage	DDB2	Damage Specific DNA Binding Protein 2	0.011	0.67
	DNMT1	DNA Methyltransferase 1	0.0043	-1.07
	ORC2	Origin Recognition Complex Subunit 2	0.028	2.08
	POLA1	DNA Polymerase Alpha 1	0.0024	-5.84
	PRIM1	DNA Primase Subunit 1	0.034	-3.70
Cell cycle	CDK6	Cyclin-dependent Kinase 6	0.0061	-2.35
	RAD21	RAD21 Cohesin Complex Component	0.016	-1.59
	KIF23	Kinesin Family Member 23	0.017	-0.64
	PDS5B	PDS5 Cohesin-associated Factor B	0.043	0.84
	SUN2	Sad1 and UNC84 Domain Containing 2	0.010	-1.55
RNA metabolism	NCBP1	Nuclear Cap Binding Protein Subunit 1	0.0077	0.66
	PRKCD	Protein Kinase C Delta	0.018	5.51
	SNRPE	Small Nuclear Ribonucleoprotein Polypeptide E	0.016	-2.83
	SF3A2	Splicing Factor 3a Subunit 2	0.019	0.78
	GEMIN2	Gem Nuclear Organelle Associated Protein 2	1.33E-05	0.97
Membrane trafficking	CLU	Clusterin	3.23E-05	-1.03
	PPP1R10	Protein Phosphatase 1 Regulatory Subunit 10	0.00090	6.53
	BAG3	BAG Cochaperone 3	0.0012	1.19
	EXOC6B	Exocyst Complex Component 6B	0.019	-5.52
	TMED3	Transmembrane p24 Trafficking Protein 3	0.031	0.59

Supp Table 2. Significantly perturbed pathways in A549/DDP cells treated with cisplatin causing cytotoxicity.

Pathway	Gene Name	Protein Name	p-value	Log2FC
Regulation by TP53	CASP6	Caspase 6	0.0075	2.56
	CHD4	Chromodomain Helicase DNA Binding Protein 4	0.032	0.88
	COX6B1	Cytochrome c Oxidase Subunit 6B1	0.0096	1.02
	ERCC2	ERCC Excision Repair 2	0.0047	3.30
	GTF2F2	General Transcription Factor IIF Subunit 2	0.049	2.36
DNA damage	BAK1	BCL2 Antagonist/Killer 1	0.020	3.54
	CBL	Cbl Proto-oncogene	0.036	2.99
	CETN1	Centrin 1	0.033	4.30
	CRIP1	Cysteine-rich protein 1	0.015	0.71
	FEN1	Flap Structure-specific Endonuclease 1	0.0013	1.60
Cell cycle	CDC27	Cell Division Cycle 27	0.0015	3.50
	CDK2	Cyclin-dependent Kinase 2	0.0078	4.62
	CDK6	Cyclin-dependent Kinase 6	0.0038	3.58
	CDKN1B	Cyclin-dependent Kinase Inhibitor 1B	0.010	3.85
	DYNC1I2	Dynein Cytoplasmic 1 Intermediate Chain 2	0.026	-0.76
RNA metabolism	CDC5L	Cell division cycle 5-like protein	0.021	1.59
	DDX1	DEAD-box Helicase 1	0.00030	5.23
	FAU	FAU Ubiquitin-like and Ribosomal protein S30 Fusion	0.0013	7.21
	GTF2F2	General Transcription Factor IIF Subunit 2	0.049	2.36
	GTF2H3	General Transcription Factor IIH Subunit 3	0.020	-1.41
Membrane trafficking	AP2A2	Adaptor-related Protein Complex 2 Subunit Alpha	0.0080	-0.74
	BIN1	Bridging Integrator 1	0.0011	2.24
	APP	Amyloid Beta Precursor Protein	0.0081	3.78
	CAPZB	Capping Actin Protein of Muscle Z-line Subunit Beta	0.047	-0.60
	CUX1	Cut Like Homeobox 1	0.0029	-1.54

Supp Table 3. Significantly perturbed pathways in A549 cells treated with AgNPs causing cytotoxicity.

Pathway	Gene Name	Protein Name	p-value	Log2FC
VEGF signaling pathway	ABL1	Tyrosine-protein Kinase ABL1	0.034	2.79
	FAS	Fas Cell Surface Death Receptor	0.0011	-2.72
	ARF4	ADP Ribosylation Factor 4	0.046	-1.76
	CFL1	Cofilin 1	0.0013	-0.67
	AP2S1	Adaptor-related Protein Complex 2 Subunit Sigma 1	0.046	2.15
Regulation by TP53	COX5B	Cytochrome C Oxidase Subunit 5B	0.00035	-6.98
	COX6B1	Cytochrome C Oxidase Subunit 6B1	0.0053	-7.82
	MTOR	Mechanistic Target of Rapamycin Kinase	0.0026	-2.15
	SFN	Stratifin	0.0082	0.67
	GPX2	Glutathione Peroxidase 2	1.99E-06	-6.10
Cell cycle	ATRX	ATRX Chromatin Remodeler	0.042	-1.14
	CCNB1	Cyclin B1	0.00097	1.62
	CCNH	Cyclin H	0.046	1.77
	CDK1	Cyclin-dependent kinase 1	0.021	0.99
	CDK2	cyclin-dependent kinase 2	2.01E-06	-5.97
Metabolism of RNA	ADAR	Adenosine-deaminase Acting on RNA	0.024	-0.748
	CLNS1A	Chloride Nucleotide-sensitive Channel 1A	0.010	0.79
	CSTF1	Cleavage Stimulation Factor Subunit 1	0.0052	5.25
	DDX1	DEAD-box Helicase 1	0.019	-0.86
	GLE1	GLE1 RNA Export Mediator	0.016	-1.82
Membrane trafficking	CUX1	Cut Like Homeobox 1	0.047	-1.70
	CTSC	Cathepsin C	0.0014	-4.71
	CD55	Complement Decay-accelerating Factor CD55	0.033	-1.18
	GOLGA1	Golgin A1	0.0011	-3.75
	IGF2R	Insulin-like Growth Factor 2 Receptor	9.75E-06	-2.29

Supp Table 4. Significantly perturbed pathways in A549/DDP cells treated with AgNPs causing cytotoxicity.

Pathway	Gene Name	Protein Name	p-value	Log2FC
VEGF signaling pathway	ARF4	ADP Ribosylation Factor 4	0.025	-0.82
	RHOC	Ras Homolog Family Member C	0.016	1.70
	CNP	2',3'-cyclic Nucleotide 3' Phosphodiesterase	0.026	0.73
	CRK	CRK Proto-oncogene, Adaptor Protein	0.049	0.92
	FN1	Fibronectin 1	0.045	-1.53
Metabolism of RNA	POLR2A	RNA Polymerase II Subunit A	0.0054	1.63
	PSMA3	Proteasome 20S Subunit Alpha 3	0.0023	0.70
	PSMD9	Proteasome 26S Subunit, Non-ATPase 9	0.029	-2.57
	PSMD10	Proteasome 26S Subunit, Non-ATPase 10	0.0050	-0.85
	RPL13	Ribosomal Protein L13	0.025	1.63
Cell cycle	AKT2	AKT Serine/threonine Kinase 2	0.038	0.86
	CKS1B	CDC28 Protein Kinase Regulatory Subunit 1B	0.016	-6.12
	KIF2A	Kinesin Family Member 2A	0.043	-0.61
	MCM3	Minichromosome Maintenance Complex Component 3	0.036	-0.93
	MCM7	Minichromosome Maintenance Complex Component 7	0.047	-3.15
Programmed cell death	HMGB2	High Mobility Group Box 2	0.021	-2.21
	TRAF2	TNF Receptor-associated Factor 2	0.015	-2.45
	STK26	Serine/threonine Kinase 26	0.010	-4.10
	CYCS	Cytochrome C, Somatic	0.037	-0.80
	CRK	CRK Proto-oncogene, Adaptor Protein	0.0488101	0.918012
Membrane trafficking	ATP1B1	ATPase Na+/K+ Transporting Subunit Beta 1	0.00071	-6.14
	ATP5F1D	ATP Synthase F1 Subunit Delta	0.00065	-1.16
	ATP6V1B2	ATPase H+ Transporting V1 Subunit B2	0.031	0.59
	COX4I1	Cytochrome C Oxidase Subunit 4I1	0.044	0.88
	COX1	Cytochrome C Oxidase Subunit I	0.017	-1.87
